# Total Syntheses of Marine Natural Products Lyngbyabellin O and Lyngbyabellin P

**DOI:** 10.3390/md23090340

**Published:** 2025-08-26

**Authors:** Jing Chen, Shiyu Li, Chao Xu, Tao Ye

**Affiliations:** 1 State Key Laboratory of Chemical Oncogenomics, Peking University Shenzhen Graduate School, Shenzhen 518055, China; chenjingchey@pku.edu.cn; 2 School of Pharmacy and Food Engineering, Wuyi University, Jiangmen 529020, China; 13991563951@163.com

**Keywords:** total synthesis, marine natural products, lyngbyabellin O, lyngbyabellin P

## Abstract

Lyngbyabellins O and P are complex natural products derived from non-ribosomal peptide synthetase/polyketide synthase (NRPS/PKS) biosynthetic pathways and have been isolated from marine cyanobacterial sources. Both metabolites are characterized by the presence of two thiazole rings and a distinctive dichlorinated *β*-hydroxy acid side chain. Notably, lyngbyabellin P is further distinguished by the incorporation of a (3*R*,4*S*)-statine moiety. Herein, we report the first total syntheses of lyngbyabellins O and P, which are achieved through the convergent coupling of three key synthetic fragments, namely, two enantiomerically enriched thiazole subunits and a hydroxycarboxylic acid derivative, the latter constructed via a stereoselective aldol reaction. The total syntheses were completed in 12 and 13 longest linear steps (LLSs) for lyngbyabellins O and P, respectively, furnishing the natural products in overall yields of 5.6% and 2.5%.

## 1. Introduction

Cyanobacteria, often implicated in harmful algal blooms, are prolific producers of structurally diverse secondary metabolites characterized by a broad spectrum of potent bioactivities. These include enzyme inhibitors, antifouling agents, and pharmacologically relevant compounds with demonstrated anticancer, antibiotic, and antiparasitic properties [1,2]. Marine organisms have long served as a valuable reservoir for pharmacological discovery, particularly in the search for bioactive compounds targeting neglected tropical diseases (e.g., malaria) [3] and addressing ecological challenges, such as marine biofouling, for which the development of environmentally benign antifouling technologies remains an urgent priority [4,5]. Systematic bioprospecting of cyanobacteria holds considerable promise for the identification and isolation of novel bioactive metabolites with therapeutic potential. Notably, the recently established genus Okeania gen. nov., delineated from Lyngbya based on phylogenetic analyses, exemplifies the taxonomic refinement within this group and was first described by Engene et al. (2013) [6].

**Figure 1:**
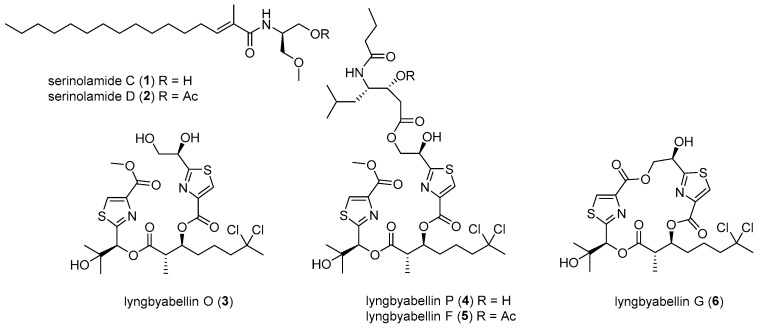
Chemical structures of serinolamides C and D and lyngbyabellins O, P, F, and G.

In 2017, Petitbois et al. reported the isolation of four novel secondary metabolites—serinolamide C (**1**), serinolamide D (**2**), lyngbyabellin O (**3**), and lyngbyabellin P (**4**)—alongside the previously characterized compounds lyngbyabellin F (**5**) and lyngbyabellin G (**6**), from a marine cyanobacterial strain of *Okeania* sp. collected in the Red Sea (Figure 1) [7]. Structural elucidation was accomplished through comprehensive nuclear magnetic resonance (NMR) spectroscopy and mass spectrometry (MS)-guided fractionation of the crude extract. The absolute configuration of lyngbyabellin O (**3**) was established via chiral-phase high-performance liquid chromatography (HPLC) analysis of its degradation products, whereas the stereochemical assignment of lyngbyabellin P (**4**) was achieved through comparative analysis with both lyngbyabellin O (**3**) and lyngbyabellin F (**5**). Bioassay-guided evaluation revealed that lyngbyabellins O (**3**) and P (**4**) exhibited pronounced antifouling activity, effectively inhibiting the larval settlement of *Amphibalanus amphitrite* with EC_50_ values of 0.24 and 0.62 µM, respectively. Furthermore, these two compounds displayed distinct cytotoxic profiles against MCF7 breast cancer cells, with GI_50_ values of 160 µM for lyngbyabellin O (3) and 9 µM for lyngbyabellin P (4), respectively.

Lyngbyabellins O (**3**) and P (**4**) share a conserved molecular framework characterized by two thiazole rings and a distinctive dichlorinated octanoic acid moiety. Notably, lyngbyabellin P (**4**) further incorporates a (3*R*,4*S*)-statine residue as a side chain. The unique structural features and compelling biological activities of these marine-derived natural products, in conjunction with their scarce natural abundance and the logistical challenges associated with their recollection, have prompted our pursuit of their total syntheses. Herein, we report our synthetic endeavors directed toward the total syntheses of lyngbyabellins O (**3**) and P (**4**).

**Figure 2:**
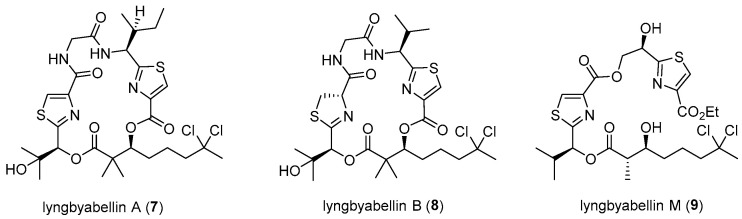
Chemical structures of lyngbyabellins A, B, and M.

**Scheme 1:**
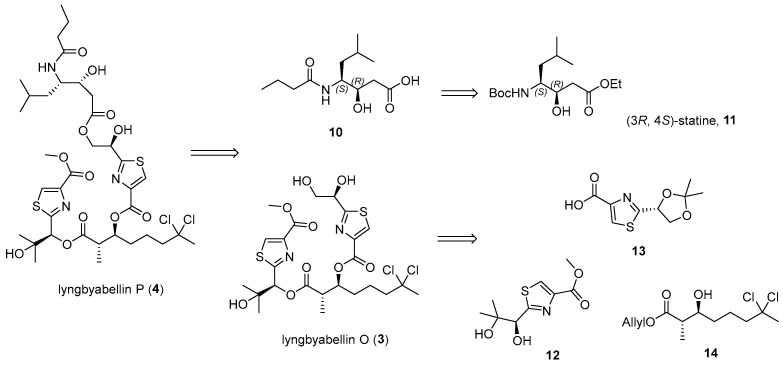
Retrosynthetic analysis of lyngbyabellins O (**3**) and P (**4**).

To the best of our knowledge, apart from the methods for the synthesis of lyngbyabellin O briefly disclosed in our patent application, [8] no other research groups have, to date, reported the total syntheses of lyngbyabellins O (**3**) and P (**4**). However, several structurally related congeners within this family—including lyngbyabellins A (**7**) [9,10,11], B (**8**) [10], and M (**9**) [12]—have been successfully synthesized (Figure 2). Our retrosynthetic analysis of lyngbyabellins O (**3**) and P (**4**) is delineated in Scheme 1. Conceptually, lyngbyabellin P (**4**) may be disassembled into lyngbyabellin O (**3**) and a (3*R*,4*S*)-statine-containing side-chain fragment (**10**). Lyngbyabellin O (**3**), in turn, can be further deconstructed into two thiazole–carboxylic acid fragments (**12** and **13**) and a dichlorinated hydroxyloctanoic acid moiety (**14**), which are envisioned to be joined via esterification reactions. This retrosynthetic approach thereby reduces the synthetic challenge to the preparation of four chiral fragments—**10**, **12**, **13**, and **14**.

## 2. Results and Discussion

**Scheme 2:**
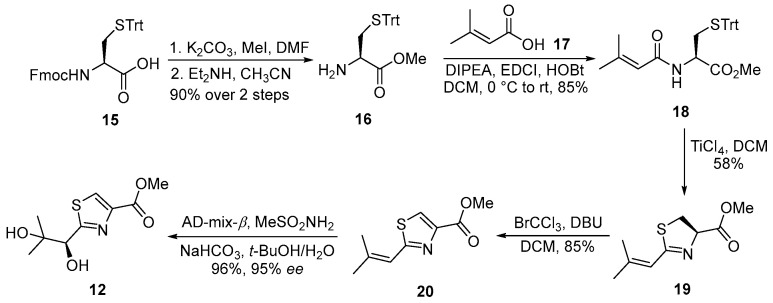
Synthesis of thiazole fragment **12**.

Our synthetic campaign commenced with intermediate **12** (Scheme 2). *N*-Fmoc-*S*-trityl-L-cysteine (**15**) was first subjected to methyl esterification using potassium carbonate (K_2_CO_3_) and methyl iodide (MeI), followed by Fmoc deprotection under basic conditions to furnish amine **16**. Subsequent coupling of amine **16** with 3,3-dimethylacrylic acid (**17**), mediated by standard peptide coupling reagents, provided compound **18**. A tandem deprotection–dehydrocyclization sequence promoted by titanium tetrachloride (TiCl_4_) then delivered thiazoline **19** in 58% yield [13]. Oxidative dehydrogenation of thiazoline **19** employing 1,8-diazabicyclo[5.4.0]undec-7-ene (DBU) and bromotrichloromethane (BrCCl_3_) afforded the corresponding thiazole **20** in 85% isolated yield [14]. Finally, thiazole **20** underwent Sharpless asymmetric dihydroxylation in the presence of methanesulfonamide and AD-mix-*β*, furnishing the desired dihydroxylated thiazole **12** in 96% yield with 95% enantiomeric excess (*ee*) [15].

**Scheme 3:**
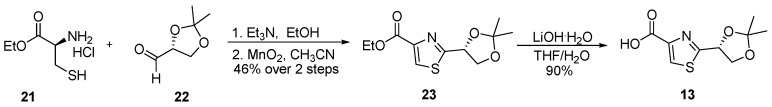
Synthesis of thiazole fragment **13**.

The chiral thiazole fragment **13** (Scheme 3) was prepared through a condensation reaction between (*S*)-ethyl cysteine hydrochloride (**21**) and (*R*)-isopropyl glyceraldehyde (**22**), followed by oxidation of the resulting thiazoline intermediate using manganese dioxide in accordance with Iwakawa’s protocol, thereby affording the 3,4-disubstituted thiazole **23** [16]. Subsequent hydrolysis of the ethyl ester furnished the target fragment **13** in an overall yield of 41%.

**Scheme 4:**
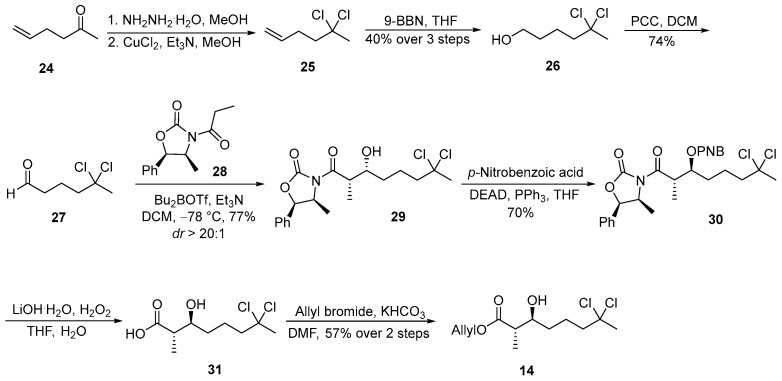
Synthesis of dichlorohydroxyl acid **14**.

The synthesis of dichlorinated hydroxyoctanoic acid **14** was accomplished as delineated in Scheme 4. The synthetic sequence commenced with 5-hexen-2-one (**24**), which was converted into the corresponding hydrazone intermediate via condensation with hydrazine hydrate, in accordance with Takeda’s protocol. Subsequent copper(II) chloride-mediated oxidative chlorination furnished gem-dichloroalkene **25** [17]. The terminal olefin of **25** was then subjected to a hydroboration–oxidation sequence, and the resulting primary alcohol **26** was further oxidized to yield dichloroaldehyde **27**. Construction of the carboxylic acid moiety was achieved through a highly *syn*-selective asymmetric aldol reaction employing propionamide **28**, derived from Evans’ chiral auxiliary, followed by a Mitsunobu inversion. Specifically, at −78 °C, enolization of **28** was affected using dibutylboron triflate (Bu_2_BOTf) and triethylamine (Et_3_N), providing *syn*-aldol adduct **29** [18]. The secondary alcohol in **29** was then inverted via a Mitsunobu reaction employing *p*-nitrobenzoic acid, diethyl azodicarboxylate (DEAD), and triphenylphosphine (PPh_3_), affording compound **30** with the desired stereochemistry at the hydroxyl-bearing carbon [19,20]. Final removal of the Evans auxiliary, followed by protection of the resultant carboxylic acid as its allyl ester, furnished the target dichlorinated hydroxyoctanoic acid **14**.

**Scheme 5:**
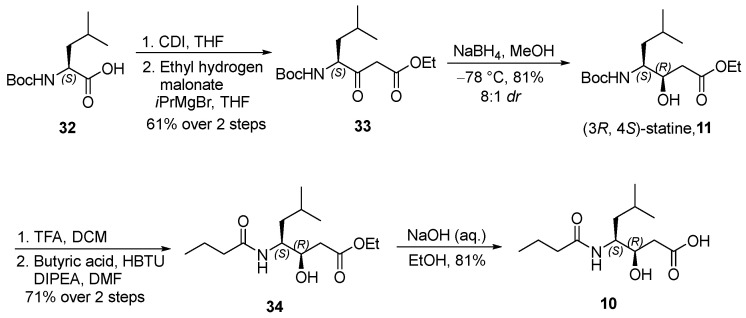
Synthesis of compound **10**.

The synthesis of fragment **10** commenced from Boc-L-leucine (**32**) (Scheme 5). The initial activation of the carboxylic acid was achieved via formation of the corresponding acyl imidazole using carbonyl diimidazole (CDI), which subsequently underwent condensation to furnish *β*-ketoester **33**. Chemoselective reduction of the ketone moiety with sodium borohydride (NaBH_4_) afforded alcohol **11** with a diastereomeric ratio of 8:1 [21]. Subsequent removal of the Boc protecting group enabled amide bond formation with *n*-butyric acid, yielding compound **34**. Final hydrolysis of the ethyl ester under basic conditions provided the target side chain fragment **10**.

**Scheme 6:**
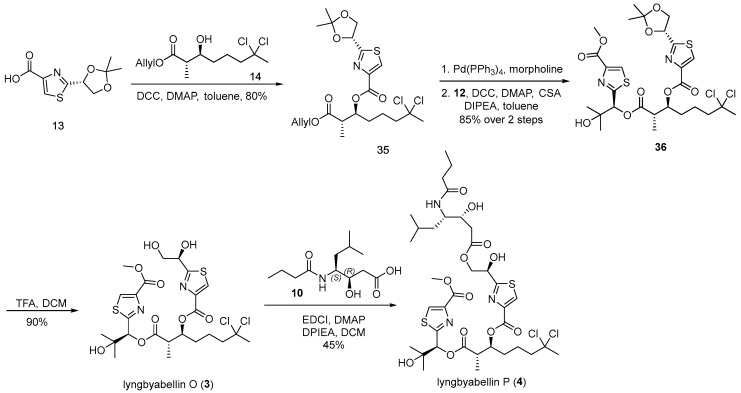
Total syntheses of lyngbyabellin O (**3**) and lyngbyabellin P (**4**).

With all four key fragments in hand, we proceeded to construct the target structures of lyngbyabellin O (**3**) and lyngbyabellin P (**4**) (Scheme 6). Thiazole carboxylic acid **13** was first coupled with alcohol **14** via DCC/DMAP-mediated esterification to furnish intermediate **35**. Subsequent removal of the allyl protecting group using Pd(PPh_3_)_4_ and morpholine enabled a second esterification with alcohol **12**, affording compound **36**. Acidic deprotection of the isopropylidene group then delivered the natural product lyngbyabellin O (**3**). Finally, esterification of compound **3** with fragment **10** completed the total synthesis of lyngbyabellin P (**4**).

A comparison of the NMR data for the synthetic samples with those reported in the original isolation study for lyngbyabellins O and P revealed an almost complete concordance, with the exception of the C-10 and C-13 resonances in lyngbyabellin O (see the Supporting Information for details) [7]. To reconcile these inconsistencies, we undertook an extensive two-dimensional NMR spectroscopic analysis of the synthetic samples. Critical HMBC correlations—specifically, from H-12 to C-13 and from H-3 to C-10—provided unequivocal validation of our structural assignment, thereby establishing the resonances at 160.9 ppm and 173.5 ppm as corresponding to C-10 and C-13, respectively. By contrast, the initial isolation study reported weaker signals at 160.9 ppm and 173.5 ppm, while assigning C-10 and C-13 instead to resonances at 166.4 ppm and 179.7 ppm, assignments made in the absence of corroborating HMBC correlations. Importantly, our chemical shift designations for C-10 (160.9 ppm) and C-13 (173.5 ppm) are in close agreement with those reported for lyngbyabellin P (C-10: 160.9 ppm; C-13: 173.0 ppm), further substantiating the accuracy of our interpretation. Taken together, the observed discrepancies between our synthetic data and the original report are most reasonably attributed to (i) the extremely limited quantity of natural material available for analysis and (ii) the likelihood of adventitious impurities. These considerations strongly suggest that the previously assigned resonances for C-10 and C-13 may, in fact, have arisen from extraneous species rather than from the natural product itself.

## 3. Materials and Methods

### 3.1. General

All reactions were conducted in flame-dried or oven-dried glassware under an atmosphere of dry nitrogen or argon. Oxygen and/or moisture-sensitive solids and liquids were transferred appropriately. The concentration of solutions in vacuo was accomplished using a rotary evaporator fitted with a water aspirator. Residual solvents were removed under a high vacuum (0.1–0.2 mm Hg). All reaction solvents were purified before use. Tetrahydrofuran (THF) was distilled from Na/benzophenone. Toluene was distilled over molten sodium metal. Dichloromethane (DCM) and trimethylamine (Et_3_N) were distilled from CaH_2_. Methanol (MeOH) was distilled from Mg/I_2_. The reagents were purchased at the highest commercial quality and used without further purification unless otherwise stated. Flash column chromatography was performed using the indicated solvents on silica gel 60 (230–400 mesh ASTM E. Qingdao, Tsingtao, China). The reactions were monitored using thin-layer chromatography (TLC), which was carried out using pre-coated sheets (silica gel 60-F250, 0.2 mm, Qingdao Haiyang Chemical Co.,Ltd., Qingdao city, China). Compounds were visualized with UV light, iodine, and ceric ammonium molybdate stainer phosphomolybdic acid in EtOH. ^1^H NMR spectra were recorded on Avance 400 MHz and Avance 500 MHz spectrometers (Bruker, Karlsruhe, Germany). Chemical shifts were reported in parts per million (ppm), relative to either a tetramethylsilane (TMS) internal standard or the signals due to the solvent. The following abbreviations are used to describe the spin multiplicity: s = singlet, d = doublet, t = triplet, q = quartet, qn = quintet, m = multiplet, br = broad, dd = doublet of doublets, dt = doublet of triplets, dq = doublet of quartets, and ddd = doublet of doublet of doublets. Other combinations are derived from those listed above. Coupling constants (J) are reported in Hertz (Hz) for corresponding solutions, and chemical shifts are reported as parts per million (ppm) relative to residual CHCl_3_ δH (7.26 ppm). ^13^C-NMR nuclear magnetic resonance spectra were recorded at 100 MHz, 125 MHz, or 150 MHz for the corresponding solutions, and chemical shifts are reported as parts per million (ppm) relative to residual CDCl_3_ δC (77.16 ppm). High-resolution mass spectra were measured on an ABI Q-star Elite (Beijing, China). Optical rotations were recorded on a Rudolph AutoPol-I polarimeter (Shanghai, China) at 589 nm with a 50 mm cell. Data are reported as follows: specific rotation (c (g/100 mL), solvent).

### 3.2. General Experimental Procedures

#### 3.2.1. Methyl *S*-Trityl-L-cysteinate (**16**)

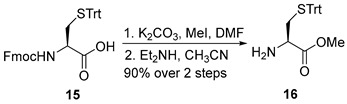



To a stirred solution of *N*-(((9H-fluoren-9-yl)methoxy)carbonyl)-*S*-trityl-D-cysteine (**15**) (5.0 g, 8.5 mmol, 1.0 equiv.) in DMF (100 mL, 0.085 M) at ambient temperature was added K_2_CO_3_ (1.4 g, 10.2 mmol, 1.2 equiv.), followed by methyl iodide (MeI; 1.1 mL, 17 mmol, 2.0 equiv.). The reaction mixture was stirred at room temperature for 3 h, after which the insoluble materials were removed by filtration through a Celite^®^ pad, and the pad was washed with ethyl acetate (3 × 100 mL). The combined organic phases were concentrated under reduced pressure to yield a colorless oil, which was used directly in the subsequent step without further purification.

The crude residue was dissolved in acetonitrile (100 mL, 0.085 M), and diethylamine (Et_2_NH; 1.1 mL, 10.2 mmol, 1.2 equiv.) was added at room temperature. The resulting solution was stirred for 1 h and then concentrated under reduced pressure. The crude product was purified by flash chromatography on silica gel (hexanes/EtOAc = 3:1) to afford compound 16 as a colorless oil (2.9 g, 90% yield over two steps). TLC: R*_f_* = 0.3 (hexanes/EtOAc = 2/1), UV and PMA stain. ${[\alpha ]\;}_{\;D}^{\;18.3}$ = +24.5 (*c* 0.8, CHCl_3_). ^1^H-NMR (400 MHz, CDCl_3_) δ 7.66–6.90 (m, 15H), 3.66 (s, 3H), 3.21 (dd, *J* = 7.8, 4.8 Hz, 1H), 2.60 (dd, *J* = 12.5, 4.8 Hz, 1H), 2.48 (dd, *J* = 12.5, 7.8 Hz, 1H). ^13^C-NMR (100 MHz, CDCl_3_) δ 174.2, 144.5, 129.6, 127.9, 126.8, 66.9, 53.8, 52.2, 36.9.

#### 3.2.2. Methyl *N*-(3-Methylbut-2-enoyl)-*S*-trityl-L-cysteinate (**18**)

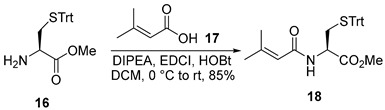



To a solution of compound **16** (500 mg, 1.3 mmol, 1.0 equiv.) in anhydrous dichloromethane (DCM, 20 mL, 0.065 M) at 0 °C was added 3-methylbut-2-enoic acid (**17**, 130 mg, 1.3 mmol, 1.0 equiv.), followed by *N*,*N*-diisopropylethylamine (DIPEA, 1.4 mL, 7.8 mmol, 6.0 equiv.), 1-ethyl-3-(3-dimethylaminopropyl)carbodiimide hydrochloride (EDCI·HCl, 498 mg, 2.6 mmol, 2.0 equiv.), and 1-hydroxybenzotriazole (HOBt, 270 mg, 2.0 mmol, 1.5 equiv.). The reaction mixture was stirred at ambient temperature for 9 h. Upon completion, the reaction mixture was concentrated under reduced pressure, and the resulting residue was dissolved in ethyl acetate (EtOAc, 50 mL). The organic phase was washed with 4% aqueous citric acid solution, and the aqueous layer was extracted with EtOAc (3 × 30 mL). The combined organic extracts were washed sequentially with saturated aqueous sodium bicarbonate solution (30 mL) and brine (30 mL), dried over anhydrous sodium sulfate (Na_2_SO_4_), filtered, and concentrated under reduced pressure. The crude product was purified by flash column chromatography on silica gel (hexanes/EtOAc = 5:1) to afford compound 18 (508 mg, 85%) as a colorless oil. TLC: R*_f_*= 0.3 (hexanes/EtOAc = 4/1), UV and PMA stain. ${[\alpha ]\;}_{\;D}^{\;17.3}$ = +4.6 (*c* 0.9, CHCl_3_). ^1^H-NMR (400 MHz, CDCl_3_) δ 7.57–7.03 (m, 15H), 5.96 (d, *J* = 7.8 Hz, 1H), 5.70–5.33 (m, 1H), 4.73 (dt, *J* = 7.9, 5.2 Hz, 1H), 3.76 (s, 3H), 2.74 (dd, *J* = 5.2, 1.1 Hz, 2H), 2.20 (d, *J* = 1.3 Hz, 3H), 1.92 (d, *J* = 1.4 Hz, 3H). ^13^C-NMR (100 MHz, CDCl_3_) δ 171.2, 166.2, 152.4, 144.3, 129.5, 128.0, 126.8, 117.8, 66.8, 52.6, 50.8, 33.9, 27.2, 19.9. HRMS (ESI) calculated for C_28_H_29_NNaO_3_S^+^ [M + Na]^+^ 482.1766, found 482.1762.

#### 3.2.3. Methyl (*R*)-2-(2-Methylprop-1-en-1-yl)-4,5-dihydrothiazole-4-carboxylate (**19**)

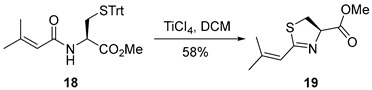



To a solution of compound **18** (400 mg, 0.87 mmol, 1.0 equiv.) in anhydrous dichloromethane (DCM, 10 mL, 0.087 M) at 0 °C under an argon atmosphere was added titanium tetrachloride (2.6 mL, 2.6 mmol, 3.0 equiv., 1.0 M in DCM). The reaction mixture was subsequently allowed to warm to room temperature and stirred for 12 h. Upon completion, the reaction was quenched with saturated aqueous sodium bicarbonate (5 mL), and the resulting aqueous phase was extracted with dichloromethane (3 × 20 mL). The combined organic extracts were washed sequentially with water (10 mL) and brine (10 mL), dried over anhydrous sodium sulfate, filtered, and concentrated under reduced pressure. The crude residue was purified by flash column chromatography on silica gel (hexanes/EtOAc = 8:1), affording compound **19** (100 mg, 58%) as a colorless oil. TLC: R*_f_* = 0.6 (hexanes/EtOAc = 8/1), UV and PMA stain. ${[\alpha ]\;}_{\;D}^{\;21.6}$ = +21.1 (*c* 1.1, CHCl_3_). ^1^H-NMR (400 MHz, CDCl_3_) δ 6.28–5.85 (m, 1H), 5.10 (t, *J* = 9.0 Hz, 1H), 3.79 (s, 3H), 3.66–3.37 (m, 2H), 2.07 (d, *J* = 1.3 Hz, 3H), 1.88 (d, *J* = 1.3 Hz, 3H). ^13^C-NMR (100 MHz, CDCl_3_) δ 171.6, 168.2, 148.5, 118.9, 77.3, 52.7, 35.3, 27.4, 20.7. HRMS (ESI) calculated for C_9_H_13_NNaO_2_S^+^ [M + Na]^+^ 222.0565, found 222.0560.

#### 3.2.4. Methyl 2-(2-Methylprop-1-en-1-yl)thiazole-4-carboxylate (**20**)

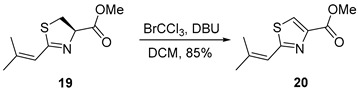



To a stirred solution of compound **19** (360 mg, 1.8 mmol, 1.0 equiv.) in anhydrous dichloromethane (DCM, 20 mL, 0.090 M) at 0 °C, bromotrichloromethane (BrCCl_3_, 0.22 mL, 2.2 mmol, 1.2 equiv.) and 1,8-diazabicyclo[5.4.0]undec-7-ene (DBU, 0.33 mL, 2.2 mmol, 1.2 equiv.) were added sequentially. The reaction mixture was gradually allowed to warm to ambient temperature and stirred for 12 h. Upon completion, the reaction was quenched with saturated aqueous ammonium chloride solution (8 mL). The resulting aqueous layer was extracted with ethyl acetate (2 × 30 mL), and the combined organic extracts were washed successively with water (30 mL) and brine (30 mL), dried over anhydrous sodium sulfate (Na_2_SO_4_), filtered, and concentrated under reduced pressure. The crude product was purified by flash column chromatography on silica gel (hexanes/EtOAc = 10:1) to afford alcohol 20 (301 mg, 85%) as a colorless oil. TLC: R*_f_* = 0.4 (hexanes/EtOAc = 8/1), UV and PMA stain. ^1^H-NMR (400 MHz, CDCl_3_) δ 8.08 (d, *J* = 0.8 Hz, 1H), 6.76–5.97 (m, 1H), 3.92 (s, 3H), 2.21–2.04 (m, 3H), 1.98 (d, *J* = 1.4 Hz, 3H). ^13^C-NMR (100 MHz, CDCl_3_) δ 166.2, 162.1, 146.2, 144.3, 126.3, 119.4, 52.4, 27.7, 20.8.

#### 3.2.5. Methyl (*S*)-2-(1,2-Dihydroxy-2-methylpropyl)thiazole-4-carboxylate (**12**)

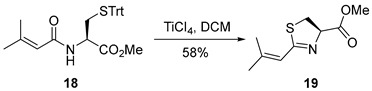



To a solution of compound **20** (500 mg, 2.5 mmol, 1.0 equiv.) in a 1:1 mixture of tert-butanol and water (20 mL each, 0.063 M) at 0 °C were sequentially added methanesulfonamide (713 mg, 7.5 mmol, 3.0 equiv.), sodium bicarbonate (1.9 g, 23 mmol, 9.0 equiv.), and AD-mix-*β* (12 g, 15 mmol, 6.0 equiv.). The reaction mixture was stirred at 0 °C for 48 h, after which it was quenched with saturated aqueous sodium sulfite solution (30 mL). The resulting aqueous phase was extracted with ethyl acetate (3 × 100 mL), and the combined organic extracts were washed with water (50 mL) and brine (50 mL), dried over anhydrous sodium sulfate, filtered, and concentrated under reduced pressure. The crude residue was purified by flash column chromatography on silica gel (hexanes/EtOAc = 5:1) to afford acid 12 as a colorless oil (555 mg, 96% yield, 95% *ee*). TLC: R*_f_* = 0.2 (hexanes/EtOAc = 1/1), UV and PMA stain. ${[\alpha ]\;}_{\;D}^{\;21}$ = −9.7 (*c* 1.1, CHCl_3_). ^1^H-NMR (400 MHz, CDCl_3_) δ 8.04 (s, 1H), 5.21 (d, *J* = 5.2 Hz, 1H), 4.77 (d, *J* = 5.1 Hz, 1H), 3.85 (s, 1H), 3.79 (s, 3H), 1.19 (s, 3H), 1.05 (s, 3H). ^13^C-NMR (100 MHz, CDCl_3_) δ 174.0, 161.9, 145.5, 128.3, 77.3, 73.0, 52.3, 25.3, 24.8. HRMS (ESI) calculated for C_9_H_13_NNaO_4_S^+^ [M + Na]^+^ 254.0463, found 254.0454.

#### 3.2.6. Ethyl (*R*)-2-(2,2-Dimethyl-1,3-dioxolan-4-yl)thiazole-4-carboxylate (**23**)

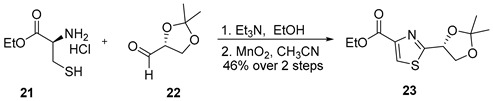



To a stirred solution of L-cysteine ethyl ester hydrochloride (**21**) (5.0 g, 27 mmol, 1.0 equiv.) and triethylamine (4.2 mL, 30 mmol, 1.1 equiv.) in ethanol (250 mL, 0.11 M) at ambient temperature was added a solution of (*R*)-(+)-glyceraldehyde acetonide (22) (3.5 g, 27 mmol, 1.0 equiv.) in ethanol (10 mL). The reaction mixture was stirred at room temperature for 5 h, after which it was concentrated under reduced pressure. The resulting residue was dissolved in diethyl ether (200 mL), and the organic phase was successively washed with water (30 mL) and brine (30 mL), dried over anhydrous Na_2_SO_4_, filtered, and concentrated in vacuo. The crude product was carried forward to the next step without further purification.

The crude intermediate was dissolved in acetonitrile (250 mL, 0.11 M) and treated with manganese dioxide (47 g, 540 mmol, 20 equiv.) at room temperature. The reaction mixture was heated to 60 °C and stirred for 48 h. Upon completion, the suspension was filtered through a pad of Celite, and the filtrate was concentrated. The crude residue was purified by flash column chromatography on silica gel (hexanes/EtOAc = 3:1) to afford compound 23 as an orange oil (3.2 g, 46% yield over two steps). TLC: R*_f_* = 0.3 (hexanes/EtOAc = 2/1), UV and PMA stain. ${[\alpha ]\;}_{\;D}^{\;31.2}$ = +15 (*c* 1.0, CHCl_3_). ^1^H-NMR (400 MHz, CDCl_3_) δ 8.13 (s, 1H), 5.42 (ddd, *J* = 6.8, 4.9, 1.8 Hz, 1H), 4.48–4.36 (m, 3H), 4.09 (ddd, *J* = 9.0, 5.0, 1.7 Hz, 1H), 1.58 (s, 3H), 1.46 (s, 3H), 1.39 (td, *J* = 7.2, 1.8 Hz, 3H). ^13^C-NMR (100 MHz, CDCl_3_) δ 172.5, 160.3, 146.5, 126.4, 110.2, 74.4, 69.4, 60.5, 25.4, 24.1, 13.4. HRMS (ESI) calculated for C_11_H_15_NNaO_4_S^+^ [M + Na]^+^ 280.0619, found 280.0612.

#### 3.2.7. (*R*)-2-(2,2-Dimethyl-1,3-dioxolan-4-yl)thiazole-4-carboxylic acid (**13**)

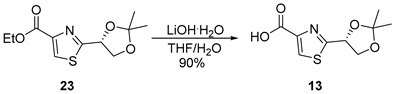



To a solution of compound **23** (2.0 g, 7.8 mmol, 1.0 equiv.) in THF/H_2_O (30 mL/15 mL, 0.17 M), LiOH·H_2_O (671 mg, 16 mmol, 2.0 equiv.) was added at 0 °C. The reaction mixture was stirred at ambient temperature for 2 h, after which it was quenched with 1 M HCl to adjust the pH to 2–3. The resulting mixture was extracted with EtOAc (3 × 100 mL), and the combined organic extracts were sequentially washed with water (50 mL) and brine (50 mL), dried over anhydrous Na_2_SO_4_, filtered, and concentrated under reduced pressure. The crude residue was purified by flash column chromatography on silica gel (DCM/MeOH = 10:1), affording acid 13 (1.6 g, 90%) as a colorless oil. TLC: R*_f_* = 0.3 (DCM/MeOH = 10/1), UV and PMA stain. ${[\alpha ]\;}_{\;D}^{\;30.2}$ = +41.1 (*c* 2.0, CHCl_3_). ^1^H-NMR (400 MHz, CDCl_3_) δ 8.27 (s, 1H), 5.45 (dd, *J* = 6.8, 5.0 Hz, 1H), 4.48 (dd, *J* = 8.9, 6.8 Hz, 1H), 4.11 (dd, *J* = 8.8, 4.9 Hz, 1H), 1.59 (s, 3H), 1.47 (s, 3H). ^13^C-NMR (100 MHz, CDCl_3_) δ 174.4, 164.9, 146.6, 129.3, 111.5, 75.4, 70.5, 26.6, 25.2. HRMS (ESI) calculated for C_9_H_11_NNaO_4_S^+^ [M + Na]^+^ 252.0306, found 252.0300.

#### 3.2.8. (4*S*,5*R*)-3-((2*S*,3*R*)-7,7-Dichloro-3-hydroxy-2-methyloctanoyl)-4-methyl-5-phenyloxazolidin-2-one (**29**)

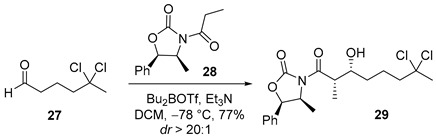



To a solution of oxazolidinone **28** (500 mg, 2.1 mmol, 1.5 equiv.) in anhydrous dichloromethane (DCM, 10 mL, 0.14 M) at −78 °C was added dibutylboron trifluoromethanesulfonate (2.2 mL, 2.2 mmol, 1.6 equiv., 1.0 M in DCM), followed by triethylamine (Et_3_N, 0.43 mL, 3.1 mmol, 2.2 equiv.). The reaction mixture was maintained at −78 °C for 30 min and then gradually warmed to 0 °C and stirred for an additional 1 h. After re-cooling the mixture to −78 °C, a solution of aldehyde **27** (235 mg, 1.4 mmol, 1.0 equiv.) in DCM (2.0 mL) was added dropwise. The resulting mixture was stirred at −78 °C for 30 min and then allowed to warm to 0 °C and stirred for a further 1.5 h. The reaction was quenched at 0 °C by the addition of 0.5 M phosphate buffer (pH 7.0, 5.0 mL), followed by methanol (12 mL) and 30% aqueous hydrogen peroxide (2.0 mL). The resulting mixture was stirred at 0 °C for 1 h, after which it was concentrated under reduced pressure. The aqueous residue was extracted with ethyl acetate (3 × 50 mL), and the combined organic extracts were washed sequentially with water (30 mL) and brine (30 mL), dried over anhydrous sodium sulfate, filtered, and concentrated in vacuo. The crude product was purified by flash column chromatography on silica gel (hexanes/EtOAc = 8:1) to afford acid **29** (441 mg, 77%) as a colorless oil, with a diastereomeric ratio (*dr*) of >20:1. TLC: R*_f_* = 0.3 (hexanes/EtOAc = 3/1), UV and PMA stain. ${[\alpha ]\;}_{\;D}^{\;24}$ = −7.48 (c 1.0, CHCl_3_). ^1^H-NMR (400 MHz, CDCl_3_) δ 7.48–7.28 (m, 5H), 5.69 (d, *J* = 7.3 Hz, 1H), 4.80 (p, *J* = 6.7 Hz, 1H), 4.00 (ddd, *J* = 9.0, 3.8, 2.7 Hz, 1H), 3.79 (qd, *J* = 7.1, 2.7 Hz, 1H), 2.37–2.19 (m, 2H), 2.15 (s, 3H), 1.91 (ddtd, *J* = 17.8, 9.8, 8.0, 7.3, 5.0 Hz, 1H), 1.74 (dddd, *J* = 15.2, 9.3, 6.1, 2.4 Hz, 1H), 1.62 (dtd, *J* = 14.5, 9.3, 5.0 Hz, 1H), 1.53–1.44 (m, 1H), 1.31–1.17 (m, 4H), 0.89 (d, *J* = 6.6 Hz, 3H). ^13^C-NMR (100 MHz, CDCl_3_) δ 177.2, 152.6, 133.1, 128.9, 128.8, 125.6, 90.6, 79.0, 71.3, 54.8, 49.6, 42.4, 37.3, 33.1, 22.5, 14.4, 10.4. HRMS (ESI) calculated for C_19_H_25_Cl_2_NNaO_4_^+^ [M + Na]^+^ 424.1058, found 424.1062.

#### 3.2.9. Allyl (2*S*,3*S*)-7,7-Dichloro-3-hydroxy-2-methyloctanoate (**14**)

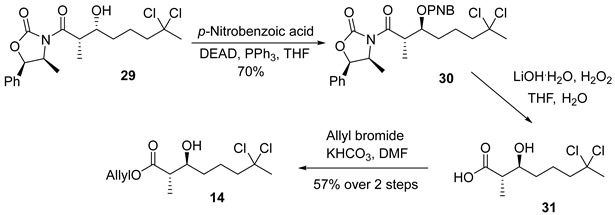



To a solution of compound **29** (400 mg, 1.0 mmol, 1.0 equiv.), triphenylphosphine (2.6 g, 10 mmol, 10 equiv.), and *p*-nitrobenzoic acid (1.0 g, 6.0 mmol, 6.0 equiv.) in anhydrous THF (20 mL, 0.05 M), a solution of diethyl azodicarboxylate (DEAD; 1.6 mL, 10 mmol, 10 equiv.) was added dropwise at room temperature. The reaction mixture was stirred for 3 h at ambient temperature, after which the solvent was removed under reduced pressure. The resulting residue was purified by flash column chromatography on silica gel (hexanes/EtOAc = 5:1) to afford ester **30** (385 mg, 70%) as a colorless oil. TLC: R*_f_* = 0.5 (hexanes/EtOAc = 3/1), UV and PMA stain.

To a solution of ester **30** (385 mg, 0.70 mmol, 1.0 equiv.) in THF/H_2_O (4 mL/1 mL, 0.14 M) at 0 °C were added 30% aqueous hydrogen peroxide (0.32 mL, 2.8 mmol, 4.0 equiv.) and lithium hydroxide monohydrate (59 mg, 1.4 mmol, 2.0 equiv.). The reaction mixture was stirred at 0 °C for 2 h, followed by the addition of aqueous sodium thiosulfate (3 mL, 0.75 M). The resulting mixture was extracted with DCM (3 × 3 mL). The combined aqueous layers were acidified to approximately pH 1 using 6 M HCl and subsequently extracted with DCM (3 × 30 mL). The combined organic extracts were washed with water (30 mL), brine (30 mL), dried over anhydrous Na_2_SO_4_, filtered, and concentrated in vacuo to yield the crude acid, which was used directly in the next step without further purification.

To a solution of the crude product in DMF (5 mL, 0.14 M) at 0 °C were added KHCO_3_ (280 mg, 2.8 mmol, 4.0 equiv.) and allyl bromide (0.24 mL, 2.8 mmol, 4.0 equiv.). The reaction mixture was allowed to warm to room temperature and stirred for 8 h. Upon completion, the reaction mixture was diluted with diethyl ether (30 mL), and the organic phase was sequentially washed with 1 M KHSO_4_ (10 mL), water (10 mL), and brine (10 mL). The organic layer was dried over anhydrous Na_2_SO_4_, filtered, and concentrated under reduced pressure. Purification by flash column chromatography on silica gel (hexanes/EtOAc = 3:1) afforded compound **14** (113 mg, 57% over 2 steps) as a colorless oil. TLC: R*_f_* = 0.3 (hexanes/EtOAc = 3/2), UV and PMA stain. ${[\alpha ]\;}_{\;D}^{\;31.2}$ = −3.3 (*c* 0.75, CHCl_3_). ^1^H-NMR (400 MHz, CDCl_3_) δ 5.92 (dddd, *J* = 17.6, 10.0, 6.1, 5.3 Hz, 1H), 5.59–5.13 (m, 2H), 4.62 (d, *J* = 5.7 Hz, 1H), 3.71 (ddd, *J* = 9.4, 6.3, 3.6 Hz, 1H), 2.57 (p, *J* = 7.0 Hz, 1H), 2.23 (dt, *J* = 10.1, 5.7 Hz, 2H), 2.15 (s, 3H), 1.90 (dtd, *J* = 12.6, 10.3, 5.1 Hz, 1H), 1.82–1.69 (m, 1H), 1.65–1.54 (m, 1H), 1.49 (ddd, *J* = 14.2, 9.4, 5.3 Hz, 1H), 1.24 (d, *J* = 7.2 Hz, 3H). ^13^C-NMR (100 MHz, CDCl_3_) δ 175.7, 132.0, 118.8, 90.7, 73.2, 65.4, 49.7, 45.4, 37.4, 34.0, 22.1, 14.5. HRMS (ESI) calculated for C_12_H_20_Cl_2_NaO_3_^+^ [M + Na]^+^ 305.0687, found 305.0681.

#### 3.2.10. Ethyl (*S*)-4-((*tert*-Butoxycarbonyl)amino)-6-methyl-3-oxoheptanoate (**33**)

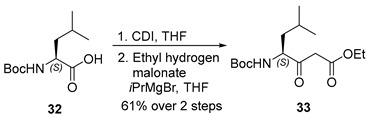



To a solution of *tert*-butoxycarbonyl-L-leucine (**32**) (2.3 g, 10.0 mmol, 1.0 equiv.) in anhydrous tetrahydrofuran (THF, 50 mL, 0.20 M) under an argon atmosphere was added *N,N′*-carbonyldiimidazole (CDI, 1.8 g, 11.0 mmol, 1.1 equiv.) at 0 °C. The resulting mixture was gradually warmed to ambient temperature and stirred for 3.5 h to generate the corresponding acyl imidazole intermediate.

Separately, a 2.2 M solution of isopropylmagnesium bromide in diethyl ether (18.2 mL, 40.0 mmol, 4.0 equiv.) was added dropwise to a solution of ethyl hydrogen malonate (2.4 mL, 20.0 mmol, 2.0 equiv.) maintained at −10 °C. The reaction mixture was subsequently allowed to warm to room temperature and stirred for 1.5 h to generate magnesium enolate. The resulting enolate solution was cooled in an ice bath, and the previously prepared imidazolide solution was added dropwise at −10 °C. The combined reaction mixture was stirred at −10 °C for 30 min and then at ambient temperature for 24 h. The reaction was quenched by the addition of 10% aqueous citric acid (10 mL), followed by further acidification to pH 3 using an additional portion of 10% aqueous citric acid (30 mL). The aqueous layer was extracted with ethyl acetate (3 × 100 mL), and the combined organic extracts were washed successively with water (50 mL) and brine (50 mL), dried over anhydrous sodium sulfate, filtered, and concentrated under reduced pressure. The crude product was purified by flash column chromatography on silica gel (hexanes/ethyl acetate = 3:1) to afford *β*-ketoester **33** (1.8 g, 61% yield over two steps) as a colorless oil. TLC: R*_f_* = 0.3 (hexanes/EtOAc = 5/1), UV and PMA stain. ${[\alpha ]\;}_{\;D}^{\;31.2}$ = −43.2 (*c* 4.1, MeOH). ^1^H-NMR (400 MHz, CDCl_3_) δ 5.07–4.89 (m, 1H), 4.43–4.26 (m, 1H), 4.15 (qd, *J* = 7.2, 1.9 Hz, 2H), 3.50 (t, *J* = 14.2 Hz, 2H), 1.67 (dd, *J* = 12.5, 5.5 Hz, 1H), 1.63–1.51 (m, 1H), 1.40 (s, 9H), 1.37–1.30 (m, 1H), 1.24 (td, *J* = 7.1, 2.0 Hz, 3H), 1.04–0.80 (m, 6H). ^13^C-NMR (100 MHz, CDCl_3_) δ 203.1, 167.1, 155.6, 80.1, 61.5, 58.3, 46.4, 39.9, 28.4, 24.9, 23.3, 21.6, 14.2. HRMS (ESI) calculated for C_15_H_27_NNaO_5_^+^ [M + Na]^+^ 324.1787, found 324.1776.

#### 3.2.11. Ethyl (3*R*,4*S*)-4-((*tert*-Butoxycarbonyl)amino)-3-hydroxy-6-methylheptanoate (**11**)

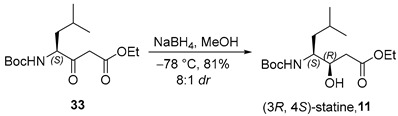



To a solution of *β*-ketoester **33** (800 mg, 2.7 mmol, 1.0 equiv.) in anhydrous methanol (25 mL, 0.11 M) under an argon atmosphere was added sodium borohydride (204 mg, 5.4 mmol, 2.0 equiv.) at −78 °C. The reaction mixture was stirred at −78 °C for 1 h, quenched with 10% aqueous citric acid (10 mL), and further acidified to pH 2 with an additional portion of 10% aqueous citric acid (20 mL). The resulting aqueous layer was extracted with ethyl acetate (3 × 50 mL), and the combined organic extracts were washed successively with water (50 mL) and brine (50 mL), dried over anhydrous sodium sulfate, filtered, and concentrated under reduced pressure. The crude product was purified by flash column chromatography on silica gel (hexanes/EtOAc = 2:1) to afford alcohol **11** (663 mg, 81%, 8:1 *dr*) as a colorless oil. TLC: R*_f_* = 0.3 (hexanes/EtOAc = 3/1), PMA stain. ${[\alpha ]\;}_{\;D}^{\;30.8}$ = −39.2 (*c* 2.6, MeOH). ^1^H-NMR (400 MHz, CDCl_3_) δ 4.71 (d, *J* = 9.1 Hz, 1H), 4.11 (q, *J* = 7.1 Hz, 2H), 3.98–3.84 (m, 1H), 3.71–3.26 (m, 1H), 2.41 (d, *J* = 7.9 Hz, 2H), 1.61 (dt, *J* = 13.6, 6.7 Hz, 1H), 1.38 (s, 9H), 1.28 (dd, *J* = 8.4, 5.9 Hz, 2H), 1.21 (t, *J* = 7.1 Hz, 3H), 0.86 (dd, *J* = 9.2, 6.6 Hz, 6H). ^13^C-NMR (100 MHz, CDCl_3_) δ 172.8, 156.2, 79.5, 71.4, 60.8, 52.8, 38.9, 38.3, 28.4, 24.8, 23.7, 21.6, 14.2. HRMS (ESI) calculated for C_15_H_29_NNaO_5_^+^ [M + Na]^+^ 326.1943, found 326.1923.

#### 3.2.12. Ethyl (3*R*,4*S*)-4-Butyramido-3-hydroxy-6-methylheptanoate (**34**)

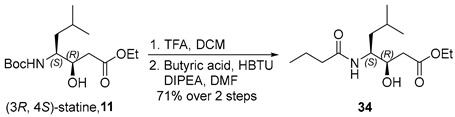



To a solution of compound **11** (300 mg, 0.99 mmol, 1.0 equiv.) in anhydrous dichloromethane (DCM, 5 mL, 0.20 M) was added trifluoroacetic acid (TFA, 2.3 mL, 30 mmol, 30 equiv.) dropwise at 0 °C. The reaction mixture was allowed to warm to ambient temperature and stirred for 2 h. Upon completion, the reaction mixture was concentrated under reduced pressure to afford an oily residue, which was carried forward to the next step without further purification.

The resulting crude residue was dissolved in *N,N*-dimethylformamide (DMF, 5 mL, 0.20 M) and cooled to 0 °C. Butyric acid (0.10 mL, 1.1 mmol, 1.1 equiv.) was then added, followed by *N,N*-diisopropylethylamine (DIPEA, 0.70 mL, 4.0 mmol, 4.0 equiv.) and *O*-(benzotriazol-1-yl)-*N,N,N′,N′*-tetramethyluronium hexafluorophosphate (HBTU, 759 mg, 2.0 mmol, 2.0 equiv.). The reaction mixture was stirred at room temperature for 9 h and subsequently concentrated in vacuo to yield a crude solid.

The crude residue was dissolved in ethyl acetate (30 mL) and washed with 4% aqueous citric acid (5 mL). The aqueous phase was extracted with ethyl acetate (3 × 20 mL), and the combined organic layers were successively washed with saturated aqueous sodium bicarbonate (15 mL) and brine (15 mL), dried over anhydrous sodium sulfate, filtered, and concentrated under reduced pressure. The crude product was purified by flash column chromatography on silica gel (hexanes/EtOAc = 1:1) to afford compound **34** (192 mg, 71% over two steps) as a white solid. TLC: R*_f_* = 0.3 (hexanes/EtOAc = 1/1), UV and PMA stain. ${[\alpha ]\;}_{\;D}^{\;30.4}$ = −22.7 (*c* 1.0, MeOH). ^1^H-NMR (400 MHz, CDCl_3_) δ 5.86 (d, *J* = 8.6 Hz, 1H), 4.12 (q, *J* = 7.2 Hz, 2H), 4.04–3.94 (m, 2H), 3.87–3.76 (m, 1H), 2.42 (d, *J* = 6.1 Hz, 2H), 2.14 (dd, *J* = 8.1, 6.8 Hz, 2H), 1.73–1.51 (m, 3H), 1.38 (ddd, *J* = 14.7, 10.7, 4.2 Hz, 1H), 1.28 (dd, *J* = 10.1, 3.5 Hz, 1H), 1.23 (t, *J* = 7.1 Hz, 3H), 0.99–0.75 (m, 9H). ^13^C-NMR (100 MHz, CDCl_3_) δ 173.7, 172.8, 71.3, 60.9, 51.6, 38.8, 38.3, 24.9, 23.7, 21.6, 19.3, 14.2, 13.8. HRMS (ESI) calculated for C_14_H_27_NNaO_4_^+^ [M + Na]^+^ 296.1838, found 296.1830.

#### 3.2.13. (3*R*,4*S*)-4-Butyramido-3-hydroxy-6-methylheptanoic acid (**10**)

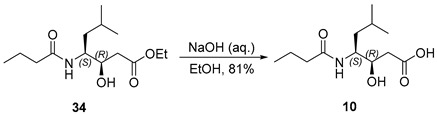



To a solution of compound **34** (200 mg, 0.73 mmol, 1.0 equiv.) in ethanol (3 mL, 0.24 M), 1 N sodium hydroxide solution (3 mL) was added dropwise. The resulting mixture was stirred at ambient temperature for 2 h. Subsequently, the ethanol was removed under reduced pressure, and the aqueous residue was diluted with water (20 mL) and cooled to 0 °C. The solution was then acidified to pH 2–3 by the careful addition of 1 N hydrochloric acid. The aqueous phase was extracted with ethyl acetate (3 × 20 mL), and the combined organic extracts were washed with brine (15 mL), dried over anhydrous sodium sulfate, filtered, and concentrated under reduced pressure. The crude product was purified by flash chromatography on silica gel using a dichloromethane/methanol solvent system (10:1) to afford compound **10** (145 mg, 81%) as a colorless oil. TLC: R*_f_* = 0.3 (DCM/MeOH = 10/1), UV and PMA stain. ${[\alpha ]\;}_{\;D}^{\;30.4}$ = −25.2 (*c* 3.3, MeOH). ^1^H-NMR (400 MHz, CDCl_3_) δ 6.47 (d, *J* = 8.5 Hz, 1H), 4.02 (t, *J* = 7.1 Hz, 2H), 2.46 (d, *J* = 6.6 Hz, 2H), 2.20 (t, *J* = 7.4 Hz, 2H), 1.61 (qd, *J* = 11.7, 11.3, 4.0 Hz, 3H), 1.48–1.36 (m, 1H), 1.35–1.26 (m, 1H), 0.92 (t, *J* = 7.2 Hz, 6H), 0.87 (d, *J* = 6.5 Hz, 3H). ^13^C-NMR (100 MHz, CDCl_3_) δ 175.8, 175.0, 71.3, 52.0, 38.6, 38.3, 25.0, 23.7, 21.6, 19.4, 13.8. HRMS (ESI) calculated for C_12_H_23_NNaO_4_^+^ [M + Na]^+^ 268.1525, found 268.1515.

#### 3.2.14. (2*S*,3*S*)-1-(Allyloxy)-7,7-dichloro-2-methyl-1-oxooctan-3-yl 2-((*R*)-2,2-di methyl- 1,3-dioxolan-4-yl)thiazole-4-carboxylate (**35**)

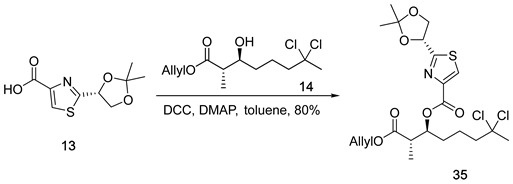



A solution of acid **13** (133 mg, 0.58 mmol, 1.1 equiv.) and alcohol **14** (150 mg, 0.53 mmol, 1.0 equiv.) in anhydrous toluene (5 mL, 0.11 M) was prepared. To this solution, dicyclohexylcarbodiimide (DCC; 132 mg, 0.64 mmol, 1.2 equiv.) and 4-dimethylaminopyridine (DMAP; 78 mg, 0.64 mmol, 1.2 equiv.) were added at 0 °C. The reaction mixture was then stirred at ambient temperature for 6 h. Upon completion, the reaction was concentrated under reduced pressure to afford a crude residue. Purification of the residue by flash chromatography on silica gel, using hexanes/ethyl acetate (10:1) as the eluent, furnished ester **35** (209 mg, 80%) as a colorless oil. TLC: R*_f_* = 0.3 (hexanes/EtOAc = 3/1), UV and PMA stain. ${[\alpha ]\;}_{\;D}^{\;21}$ = +11.4 (*c* 1.2, CHCl_3_). ^1^H-NMR (400 MHz, CDCl_3_) δ 8.01 (s, 1H), 5.75 (ddt, *J* = 17.2, 10.4, 5.8 Hz, 1H), 5.29 (dd, *J* = 6.8, 4.9 Hz, 2H), 5.17 (dq, *J* = 17.2, 1.6 Hz, 1H), 5.06 (dq, *J* = 10.4, 1.3 Hz, 1H), 4.45 (dq, *J* = 5.9, 1.3 Hz, 2H), 4.34 (dd, *J* = 8.8, 6.8 Hz, 1H), 3.99 (dd, *J* = 8.8, 4.9 Hz, 1H), 2.86 (p, *J* = 7.0 Hz, 1H), 2.18 (ddd, *J* = 15.1, 6.6, 2.9 Hz, 1H), 2.12–2.03 (m, 1H), 1.99 (s, 3H), 1.78–1.58 (m, 4H), 1.46 (s, 3H), 1.34 (s, 3H), 1.13 (d, *J* = 7.1 Hz, 3H). ^13^C-NMR (100 MHz, CDCl_3_) δ 173.5, 172.6, 160.2, 146.7, 131.8, 127.7, 118.4, 111.0, 90.1, 75.2, 74.9, 70.2, 65.2, 49.1, 43.1, 37.1, 30.4, 26.3, 24.9, 21.2, 12.8. HRMS (ESI) calculated for C_21_H_29_Cl_2_NNaO_6_S^+^ [M + Na]^+^ 516.0990, found 516.0990.

#### 3.2.15. (2*S*,3*S*)-7,7-Dichloro-1-((*S*)-2-hydroxy-1-(4-(methoxycarbonyl)thiazol-2-yl)-2- methylpropoxy)-2-methyl-1-oxooctan-3-yl 2-((*R*)-2,2-dimethyl-1,3-dioxo lan-4-yl) thiazole-4-carboxylate (**36**)

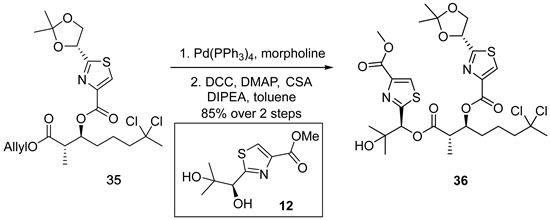



A solution of compound **35** (200 mg, 0.41 mmol, 1.0 equiv.) in anhydrous tetrahydrofuran (5 mL, 0.082 M) was treated with tetrakis(triphenylphosphine)palladium(0) (Pd(PPh_3_)_4_) (47 mg, 0.041 mmol, 0.1 equiv.) and morpholine (0.36 mL, 4.1 mmol, 10 equiv.) at ambient temperature. The reaction mixture was stirred for 15 min before being diluted with diethyl ether (30 mL). The resulting organic layer was washed sequentially with 1 M potassium bisulfate aqueous solution (5 mL) and brine (5 mL), dried over anhydrous sodium sulfate, filtered, and concentrated under reduced pressure to yield an oily residue. This residue was carried forward without further purification.

Subsequently, the crude acid thus obtained and alcohol **12** (104 mg, 0.45 mmol, 1.1 equiv.) were dissolved in anhydrous toluene (5 mL, 0.082 M). To this solution, *N,N*-diisopropylethylamine (DIPEA) (37 µL, 0.21 mmol, 0.5 equiv.), 4-dimethylaminopyridine (DMAP) (26 mg, 0.21 mmol, 0.5 equiv.), camphorsulfonic acid (CSA) (28 mg, 0.12 mmol, 0.3 equiv.), and dicyclohexylcarbodiimide (DCC) (136 mg, 0.66 mmol, 1.6 equiv.) were added at 0 °C. The reaction mixture was stirred at room temperature for 6 h and subsequently concentrated under reduced pressure to afford a crude residue. Purification by flash column chromatography on silica gel, employing dichloromethane/methanol (100:1, *v*/*v*) as eluent, furnished ester **36** (233 mg, 85% yield over two steps) as a colorless oil. TLC: R*_f_* = 0.4 (DCM/MeOH = 30/1), UV and PMA stain. ${[\alpha ]\;}_{\;D}^{\;24.8}$ = −13.9 (*c* 1.0, CHCl_3_). ^1^H-NMR (400 MHz, CDCl_3_) δ 8.15 (s, 1H), 8.14 (s, 1H), 6.11 (s, 1H), 5.41 (dd, *J* = 6.8, 4.8 Hz, 1H), 5.39–5.33 (m, 1H), 4.45 (dd, *J* = 8.9, 6.8 Hz, 1H), 4.11 (s, 1H), 4.05 (dd, *J* = 8.9, 4.9 Hz, 1H), 3.90 (s, 3H), 2.97 (qd, *J* = 7.1, 4.3 Hz, 1H), 2.23 (dt, *J* = 14.6, 7.3 Hz, 1H), 2.20–2.08 (m, 1H), 2.08 (s, 3H), 1.77 (tdd, *J* = 18.0, 7.4, 4.6 Hz, 4H), 1.56 (s, 3H), 1.42 (s, 3H), 1.33 (s, 3H), 1.24 (d, *J* = 7.1 Hz, 3H), 1.15 (s, 3H). ^13^C-NMR (100 MHz, CDCl_3_) δ 174.9, 170.9, 167.5, 161.7, 160.9, 146.7, 146.1, 128.6, 128.5, 111.3, 90.1, 78.6, 75.2, 75.0, 71.8, 70.5, 52.5, 49.1, 43.7, 37.4, 31.5, 26.8, 26.5, 25.5, 25.0, 21.7, 13.6. HRMS (ESI) calculated for C_27_H_36_Cl_2_N_2_NaO_9_S_2_^+^ [M + Na]^+^ 689.1137, found 689.1430.

#### 3.2.16. Lyngbyabellin O (**3**)

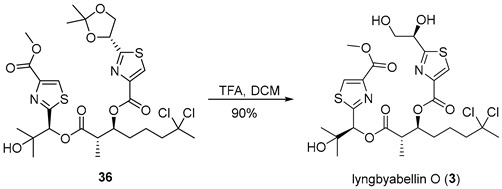



A solution of compound **36** (30 mg, 0.045 mmol, 1.0 equiv.) in dichloromethane (3 mL, 0.015 M) was cooled to 0 °C, and trifluoroacetic acid (0.11 mL, 1.4 mmol, 30 equiv.) was added dropwise. The resulting mixture was stirred at ambient temperature for 2 h, after which the solvent was removed under reduced pressure to yield a solid residue. The crude product was purified by flash chromatography on silica gel using a dichloromethane/methanol solvent system (10:1) to afford lyngbyabellin O (**3**) (26 mg, 90%) as a white solid. TLC: R*_f_* = 0.5 (DCM/MeOH = 10/1), UV and PMA stain. ${[\alpha ]\;}_{\;D}^{\;23.5}$= −11.0 (*c* 1.0, MeOH). ^1^H-NMR (400 MHz, CDCl_3_) δ 8.21 (s, 1H), 8.16 (s, 1H), 6.14 (s, 1H), 5.41 (dt, *J* = 7.7, 4.8 Hz, 1H), 5.08 (t, *J* = 4.8 Hz, 1H), 4.12–3.95 (m, 2H), 3.94 (s, 3H), 3.06–2.93 (m, 1H), 2.33–2.20 (m, 1H), 2.23–2.14 (m, 1H), 2.12 (s, 3H), 1.95–1.71 (m, 4H), 1.40 (s, 3H), 1.27 (d, *J* = 7.1 Hz, 3H), 1.18 (s, 3H). ^13^C-NMR (100 MHz, CDCl_3_) δ 173.5, 171.0, 167.1, 161.7, 160.9, 146.2, 146.0, 129.1, 128.8, 90.1, 78.3, 75.1, 72.2, 71.8, 66.0, 52.6, 49.1, 43.8, 37.4, 31.6, 26.9, 25.3, 21.6, 13.8. HRMS (ESI) calculated for C_24_H_32_Cl_2_N_2_NaO_9_S_2_^+^ [M + Na]^+^ 649.0824, found 649.0821.

#### 3.2.17. Lyngbyabellin P (**4**)

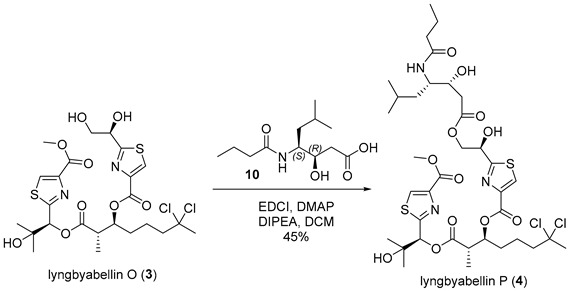



To a stirred solution of lyngbyabellin O (**3**) (40 mg, 0.064 mmol, 1.0 equiv.) and acid **10** (47 mg, 0.19 mmol, 3.0 equiv.) in anhydrous dichloromethane (3 mL, 0.021 M) at 0 °C were added 1-ethyl-3-(3-dimethylaminopropyl)carbodiimide hydrochloride (EDCI) (36 mg, 0.19 mmol, 3.0 equiv.), *N,N*-diisopropylethylamine (DIPEA) (66 µL, 0.38 mmol, 6.0 equiv.), and 4-dimethylaminopyridine (DMAP) (4.6 mg, 0.038 mmol, 0.6 equiv.). The reaction mixture was subsequently allowed to warm to ambient temperature and stirred for 6 h. Upon completion, the reaction was concentrated under reduced pressure to afford a crude solid residue. The residue was dissolved in ethyl acetate (30 mL), and the resulting solution was quenched with 4% aqueous citric acid (5 mL). The aqueous phase was separated and extracted with ethyl acetate (3 × 20 mL). The combined organic extracts were washed sequentially with saturated sodium bicarbonate solution (15 mL) and brine (15 mL), dried over anhydrous sodium sulfate, filtered, and concentrated under reduced pressure. Purification of the crude product by flash chromatography on silica gel, employing a dichloromethane/methanol mixture (10:1, *v*/*v*) as the eluent, afforded lyngbyabellin P (**4**) as a colorless amorphous solid (25 mg, 45% yield). TLC: R*_f_* = 0.3 (DCM/MeOH = 10/1), UV and PMA stain. ${[\alpha ]\;}_{\;D}^{\;30.4}$= −23.9 (*c* 1.1, MeOH). ^1^H-NMR (400 MHz, CDCl_3_) δ 8.20 (s, 1H), 8.16 (s, 1H), 6.12 (s, 1H), 5.72 (s, 1H), 5.64 (d, *J* = 8.4 Hz, 1H), 5.43 (dt, *J* = 7.8, 4.6 Hz, 1H), 5.30 (t, *J* = 5.6 Hz, 1H), 4.56 (d, *J* = 5.6 Hz, 2H), 4.18–4.07 (m, 1H), 4.07–4.01 (m, 1H), 3.94 (s, 3H), 3.03 (qd, *J* = 7.3, 5.0 Hz, 1H), 2.56 (d, *J* = 5.9 Hz, 2H), 2.32–2.13 (m, 1H), 2.23–2.15 (m, 2H), 2.12 (s, 3H), 1.93–1.74 (m, 4H), 1.74–1.55 (m, 2H), 1.63–1.55 (m, 1H), 1.54–1.42 (m, 1H), 1.37 (s, 3H), 1.38–1.29 (m, 1H), 1.29 (d, *J* = 7.1 Hz, 3H), 1.19 (s, 3H), 0.94 (t, *J* = 6.7 Hz, 3H), 0.94 (d, *J* = 6.3 Hz, 3H), 0.89 (d, *J* = 6.4 Hz, 3H). ^13^C-NMR (100 MHz, CDCl_3_) δ 174.3, 173.1, 171.9, 171.1, 167.4, 161.7, 160.9, 146.4, 146.2, 129.0, 128.6, 90.1, 78.5, 75.0, 72.0, 71.7, 69.9, 67.8, 52.5, 51.6, 49.1, 43.7, 38.8, 38.6, 37.6, 37.4, 31.3, 26.6, 25.4, 24.9, 23.6, 21.6, 21.5, 19.2, 13.7, 13.5. HRMS (ESI) calculated for C_36_H_53_Cl_2_N_3_NaO_12_S_2_^+^ [M + Na]^+^ 876.2345, found 876.2339.

## 4. Conclusions

In conclusion, we have achieved the inaugural total syntheses of lyngbyabellin O and lyngbyabellin P, compounds distinguished by their intricate structural features and notable biological activities. Our synthetic strategy encompassed the enantioselective preparation of dichlorohydroxyl carboxylic acid and statin moieties, the assembly of the thiazole heterocycle, and the implementation of efficient fragment coupling methodologies. These efforts collectively culminated in the successful construction of the targeted lyngbyabellin analogues.

## Data Availability

Copies of ^1^H and ^13^C NMR spectra, as well as associated discussion and structural assignments, are provided in the Supporting Information.

## Supplementary Materials

The following supporting information can be downloaded at https://www.mdpi.com/article/10.3390/md23090340/s1, Copies of NMR spectra (^1^H and ^13^C) of **1**–**5**, **9**, **10**, **12**, **16**, and **18**–**26**.
