# Supplementary material for: Total Syntheses of Marine Natural Products Lyngbyabellin O and Lyngbyabellin P
Source: Mar Drugs. 2025 Aug 26;23(9):340. doi: 10.3390/md23090340 (PMC12471755; doi:10.3390/md23090340)

# Supporting Information

# Total Syntheses of Marine Natural Products Lyngbyabellin O and Lyngbyabellin P

**Table of Contents**


1. NMR comparison of natural and synthetic lyngbyabellin O and lyngbyabellin P
2. Specific rotation data of natural and synthetic lyngbyabellin O and lyngbyabellin P
3. NMR spectra
4. 2D NMR correlations for the structures of synthetic lyngbyabellin O and lyngbyabellin P
5. Comparative $^{13}C$ NMR spectra of natural and synthetic lyngbyabellin O and lyngbyabellin P

1. NMR comparison of natural and synthetic lyngbyabellin O and lyngbyabellin P

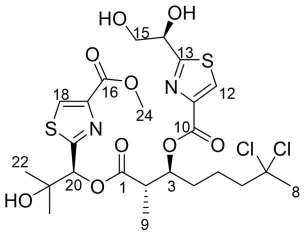


lyngbyabellin O (3)

| No. | Lyngbyabellin O | | |
|---|---|---|---|
| | **Natural ($\delta_3$)** | **Synthetic ($\delta_4$)** | **$\Delta_\delta$=$\delta_3$-$\delta_4$** |
| **1** | 171.0 | 171.0 | 0.0 |
| **2** | 43.8 | 43.8 | 0.0 |
| **3** | 75.2 | 75.1 | 0.1 |
| **4** | 31.6 | 31.6 | 0.0 |
| **5** | 21.6 | 21.6 | 0.0 |
| **6** | 49.1 | 49.1 | 0.0 |
| **7** | 90.1 | 90.1 | 0.0 |
| **8** | 37.4 | 37.4 | 0.0 |
| **9** | 13.8 | 13.8 | 0.0 |
| **10** | 166.4 | 160.9 | 5.5 |
| **11** | 146.2 | 146.2 | 0.0 |
| **12** | 129.0 | 129.1 | -0.1 |
| **13** | 179.7 | 173.5 | 5.2 |
| **14** | 71.7 | 71.8 | -0.1 |
| **15** | 66.0 | 66.0 | 0.0 |
| **16** | 161.8 | 161.7 | 0.1 |
| **17** | 146.1 | 146.0 | 0.1 |
| **18** | 128.7 | 128.8 | -0.1 |
| **19** | 167.0 | 167.1 | 0.1 |
| **20** | 78.3 | 78.3 | 0.0 |
| **21** | 72.2 | 72.2 | 0.0 |
| **22** | 25.3 | 25.3 | 0.0 |
| **23** | 26.9 | 26.9 | 0.0 |
| **24** | 52.6 | 52.6 | 0.0 |

| No. | Lyngbyabellin O | | |
|---|---|---|---|
| | **Natural ($\delta_1$)**<br>**δH (mult., *J*, Hz)** | **Synthetic ($\delta_2$)**<br>**δH (mult., *J*, Hz)** | **$\Delta_\delta$=$\delta_1$-$\delta_2$**<br>**δH (mult., *J*, Hz)** |
| **1** | | | |

| | | | |
|---|---|---|---|
| **2** | 3.00 (dq, 4.2, 6.6) | 2.99 (m) | 0.01 |
| **3** | 5.43, (dt, 4.8, 12.0) | 5.41 (dt, 4.8, 7.7) | 0.02 |
| **4** | 1.81 (m) | 1.81 (m) | 0.00 |
| **5** | 1.81 (m) | 1.81 (m) | 0.00 |
| **6** | 2.18, 2.25 (m) | 2.18, 2.25, m | 0.00 |
| **7** | | | |
| **8** | 2.12 (s) | 2.12 (s) | 0.00 |
| **9** | 1.28 (d, 6.6) | 1.27 (d, 7.1) | 0.01 |
| **10** | | | |
| **11** | | | |
| **12** | 8.24 (s) | 8.21 (s) | 0.03 |
| **13** | | | |
| **14** | 5.09 (br) | 5.08 (t, 4.8) | 0.01 |
| **15** | 4.02 (brd) | 4.02 (m) | 0.00 |
| **16** | | | |
| **17** | | | |
| **18** | 8.17 (s) | 8.16 (s) | 0.01 |
| **19** | | | |
| **20** | 6.16 (s) | 6.14 (s) | 0.02 |
| **21** | | | |
| **22** | 1.18 (s) | 1.18 (s) | 0.00 |
| **23** | 1.41 (s) | 1.40 (s) | 0.01 |
| **24** | 3.95 (s) | 3.94 (s) | 0.01 |

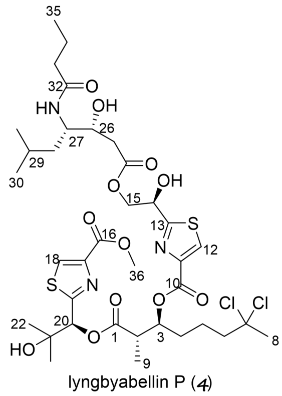


lyngbyabellin P (*4*)

| **No.** | Lyngbyabelin P | | |
|---|---|---|---|
| | **Natural ($\delta_3$)** | **Synthetic ($\delta_4$)** | **$\Delta_\delta$=$\delta_3$-$\delta_4$** |
| **1** | 171.0 | 171.1 | -0.1 |
| **2** | 43.7 | 43.7 | 0.0 |
| **3** | 75.0 | 75.0 | 0.0 |
| **4** | 31.4 | 31.3 | 0.1 |
| **5** | 21.6 | 21.6 | 0.0 |

| | | | |
|---|---|---|---|
| **6** | 49.1 | 49.1 | 0.0 |
| **7** | 90.1 | 90.1 | 0.0 |
| **8** | 37.4 | 37.4 | 0.0 |
| **9** | 13.6 | 13.5 | 0.1 |
| **10** | 160.9 | 160.9 | 0.0 |
| **11** | 146.4 | 146.4 | 0.0 |
| **12** | 129.0 | 129.0 | 0.0 |
| **13** | 173.0 | 173.1 | -0.1 |
| **14** | 70.0 | 69.9 | 0.1 |
| **15** | 67.8 | 67.8 | 0.0 |
| **16** | 161.7 | 161.7 | 0.0 |
| **17** | 146.2 | 146.2 | 0.0 |
| **18** | 128.5 | 128.6 | -0.1 |
| **19** | 167.4 | 167.4 | 0.0 |
| **20** | 78.4 | 78.5 | -0.1 |
| **21** | 72.0 | 72.0 | 0.0 |
| **22** | 26.6 | 26.6 | 0.0 |
| **23** | 25.4 | 25.4 | 0.0 |
| **24** | 171.9 | 171.9 | 0.0 |
| **25** | 37.5 | 37.6 | -0.1 |
| **26** | 71.8 | 71.7 | 0.1 |
| **27** | 51.6 | 51.6 | 0.0 |
| **28** | 39.0 | 38.8 | 0.2 |
| **29** | 25.0 | 24.9 | 0.1 |
| **30** | 23.5 | 23.6 | -0.1 |
| **31** | 21.5 | 21.5 | 0.0 |
| **32** | 174.3 | 174.3 | 0.0 |
| **33** | 38.6 | 38.6 | 0.0 |
| **34** | 19.2 | 19.2 | 0.0 |
| **35** | 13.7 | 13.7 | 0.0 |
| **36** | 52.5 | 52.5 | 0.0 |

| **No.** | Lyngbyabelin P | | |
|---|---|---|---|
| | **Natural ($\delta_1$) δH (mult., *J*, Hz)** | **Synthetic ($\delta_2$) δH (mult., *J*, Hz)** | **$\Delta_\delta=\delta_1-\delta_2$ δH (mult., *J*, Hz)** |
| **1** | | | |
| **2** | 3.03 (dq, 4.8, 7.2) | 3.03 (dq, 5.0, 7.3) | 0.00 |
| **3** | 5.43 (dt, 7.8, 4.2) | 5.43 (dt, 7.8, 4.6 ) | 0.00 |
| **4** | 1.82 (m) | 1.82 (m) | 0.00 |
| **5** | 1.79 (m) | 1.79 (m) | 0.00 |
| **6** | 2.18, 2.26 (m) | 2.17, 2.25 (m) | 0.01,0.01 |

| | | | |
|---|---|---|---|
| **7** | | | |
| **8** | 2.12 (s) | 2.12 (s) | 0.00 |
| **9** | 1.29 (d, 6.6) | 1.29 (d, 7.1) | 0.00 |
| **10** | | | |
| **11** | | | |
| **12** | 8.22 (s) | 8.20 (s) | 0.02 |
| **13** | | | |
| **14** | 5.29 (dd, 3.6, 7.8) | 5.30 (t, 5.6) | -0.01 |
| **15** | 4.55 (dd, 11.4, 3.6)<br>4.59 (dd, 11.4, 7.8) | 4.56 (d, 5.6) | -0.01, 0.03 |
| **16** | | | |
| **17** | | | |
| **18** | 8.17 (s) | 8.16 (s) | 0.01 |
| **19** | | | |
| **20** | 6.13 (s) | 6.12 (s) | 0.01 |
| **21** | | | |
| **22** | 1.38 (s) | 1.37 (s) | 0.01 |
| **23** | 1.19 (s) | 1.19 (s) | 0.00 |
| **24** | | | |
| **25** | 2.56 (d, 5.4) | 2.56 (d, 5.9) | 0.00 |
| **26** | 3.90 (ddd, 6.0, 6.0, 6.0) | 3.91 (m) | -0.01 |
| **27** | 4.07 (m) | 4.07 (m) | 0.00 |
| **27-NH** | 5.52 (d, 8.4) | 5.64 (d, 8.4) | -0.12 |
| **28** | 1.34, 1.50 (m) | 1.34, 1.48, m | 0.00, 0.02 |
| **29** | 1.63 (m) | 1.60 (m) | 0.03 |
| **30** | 0.95 (d, 6.0) | 0.94 (d, 6.3) | 0.01 |
| **31** | 0.90 (d, 6.0) | 0.89 (d, 6.4) | 0.01 |
| **32** | | | |
| **33** | 2.20 (m) | 2.20 (m) | 0.00 |
| **34** | 1.67 (m) | 1.66 (m) | 0.01 |
| **35** | 0.97 (t, 7.8) | 0.94 (t, 6.7) | 0.03 |
| **36** | 3.95 (s) | 3.94 (s) | 0.01 |

2. Specific rotation data of natural and synthetic lyngbyabellin O and lyngbyabellin P

| | | Value |
|---|---|---|
| Lyngbyabellin O | Natural | $[\alpha]_D^{26}$= -9.5 (c 0.23, MeOH) |
| | Synthetic | $[\alpha]_D^{23.5}$= -11.0 (c 1.0, MeOH) |
| Lyngbyabellin P | Natural | - |
| | Synthetic | $[\alpha]_D^{30.4}$= -23.9 (c 1.1, MeOH) |

3. NMR spectra

**Figures S1** $^{1}$H NMR Spectrum of **16** (400 MHz, $CDCl_3$)

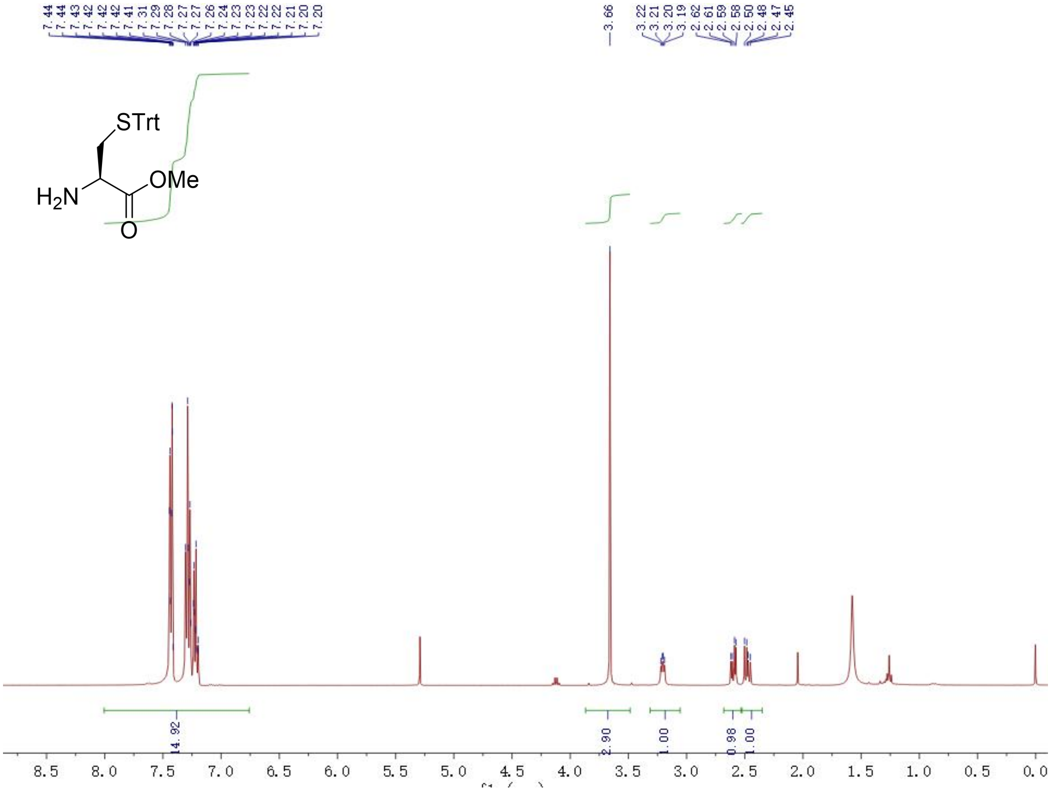

**Figures S2** $^{13}$C NMR Spectrum of **16** (101 MHz, $CDCl_3$)

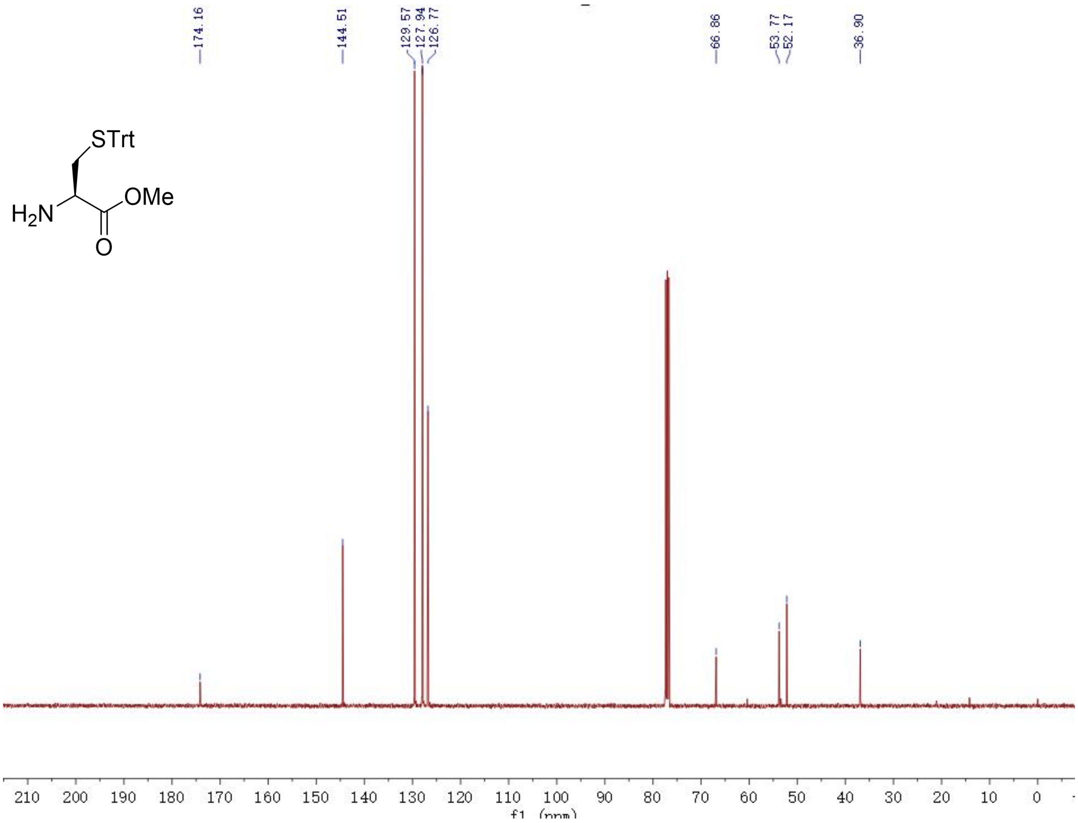

**Figures S3** $^{1}$H NMR Spectrum of **18** (400 MHz, $CDCl_3$)

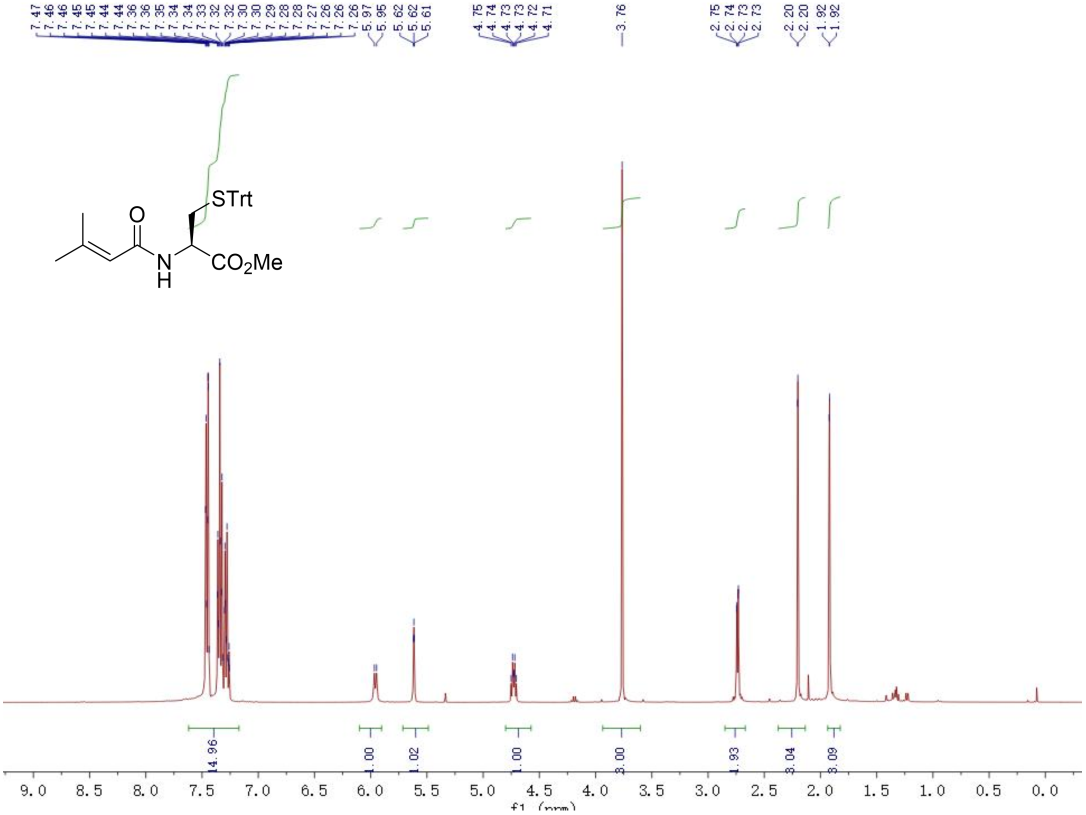


**Figures S4** $^{13}$C NMR Spectrum of **18** (101 MHz, $CDCl_3$)

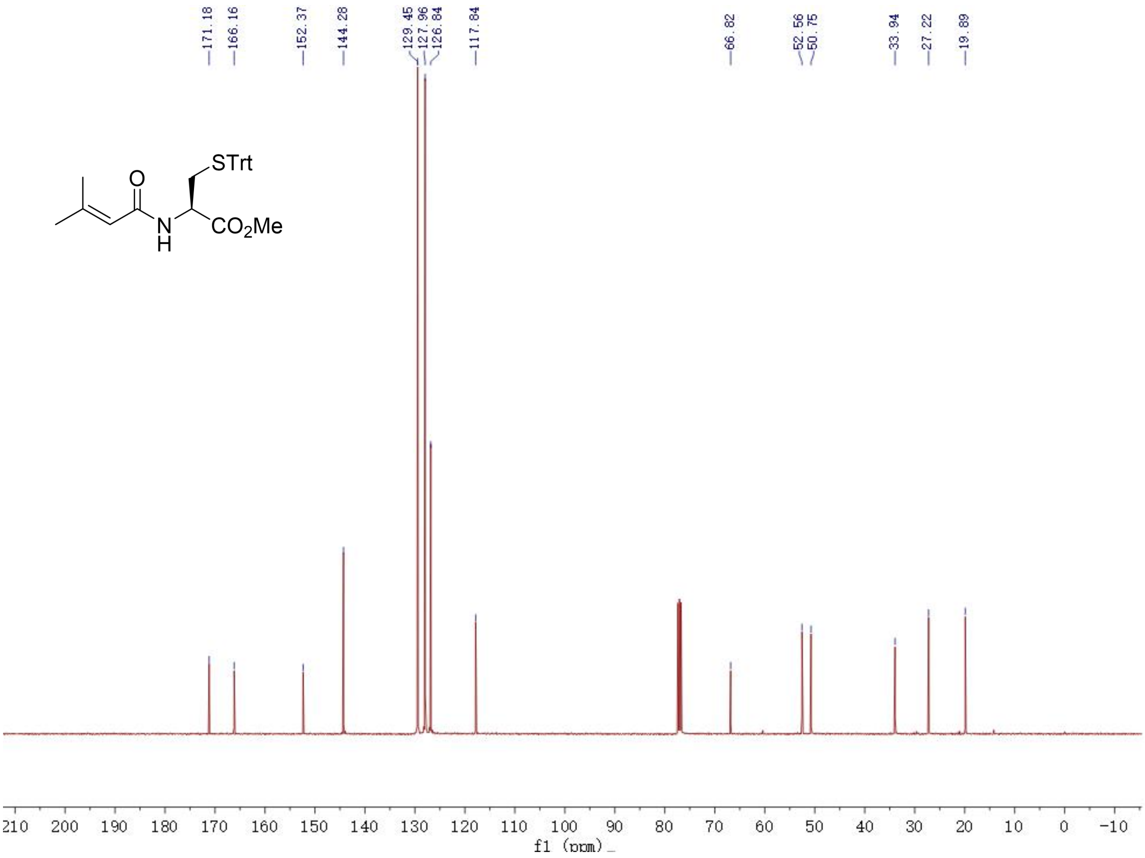

**Figures S5** $^{1}$H NMR Spectrum of **19** (400 MHz, $CDCl_3$)

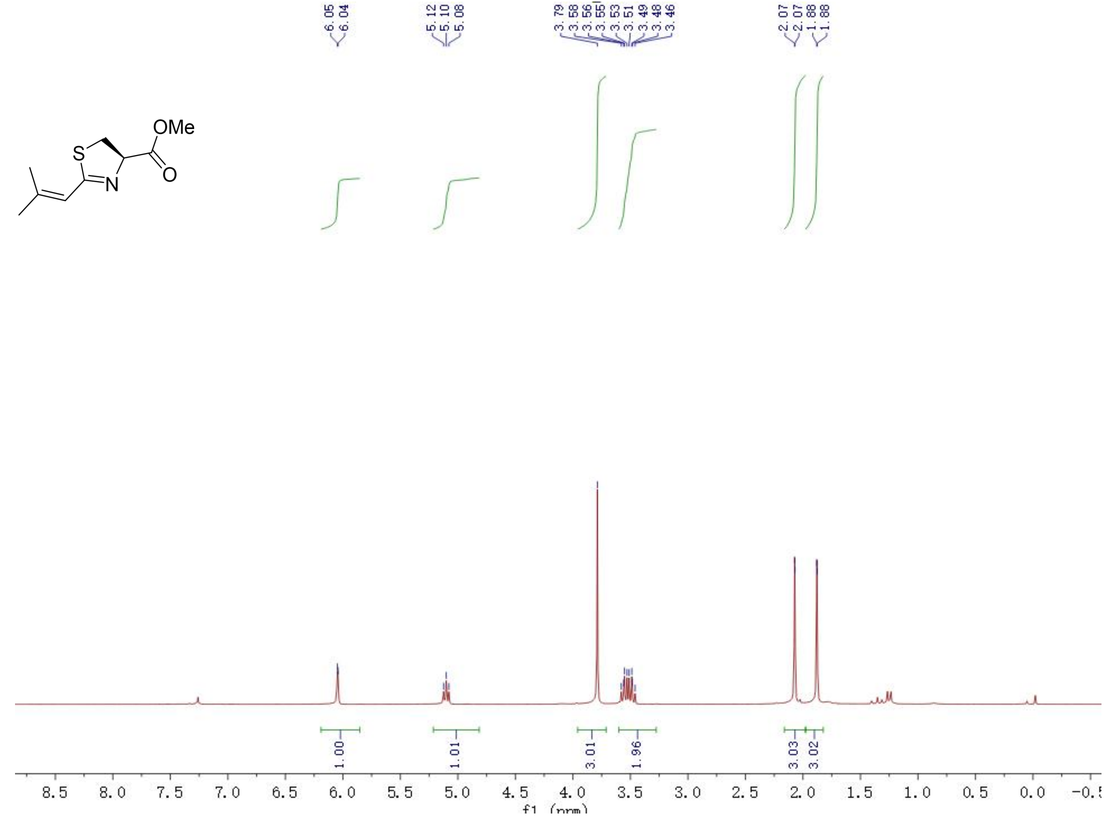


**Figures S6** $^{13}$C NMR Spectrum of **19** (101 MHz, $CDCl_3$)

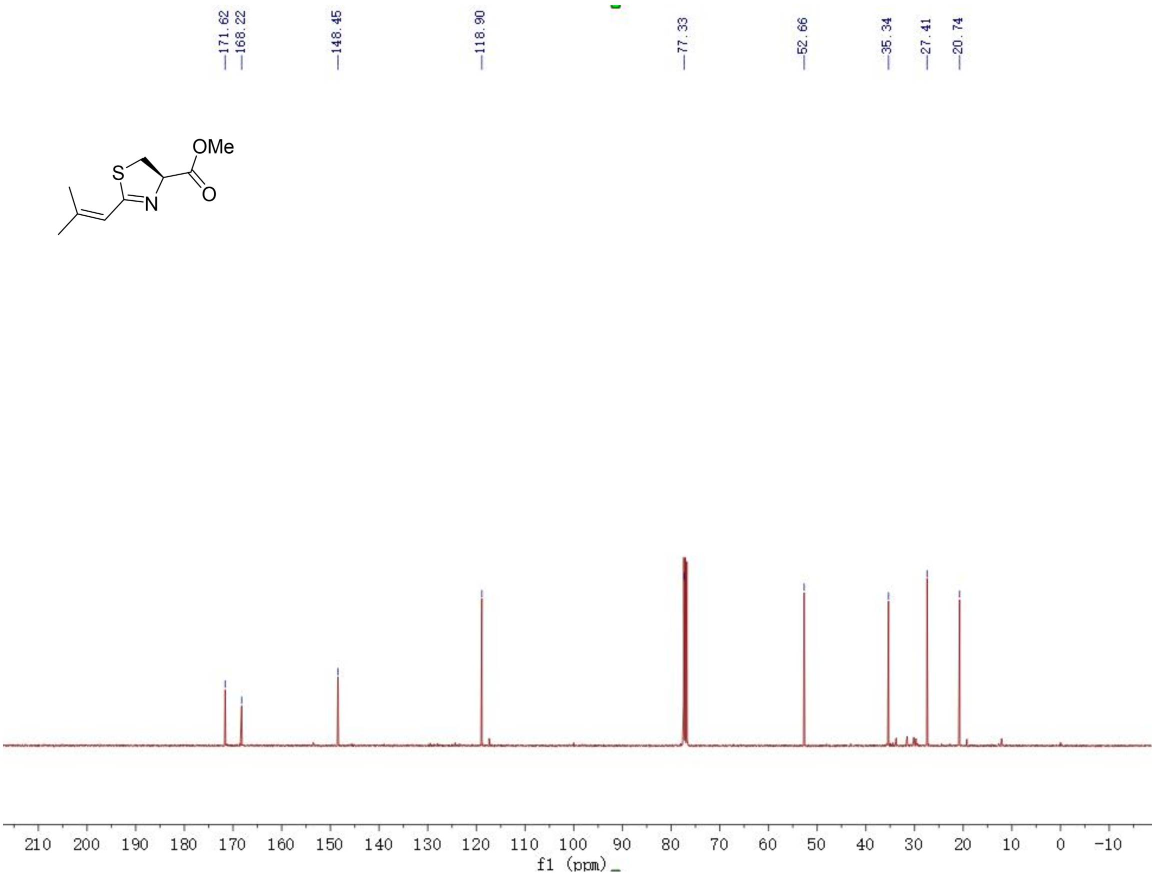

**Figures S7** $^{1}$H NMR Spectrum of **20** (400 MHz, $CDCl_3$)

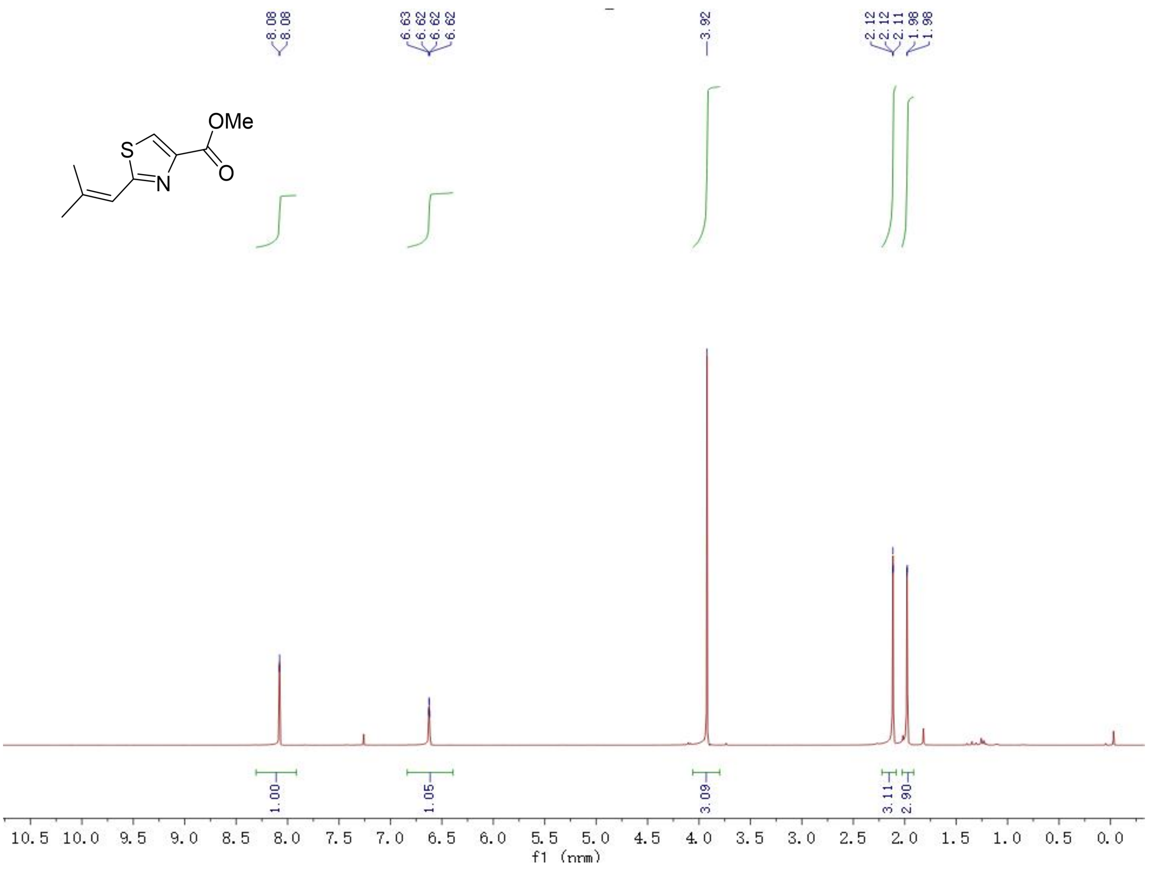

**Figures S8** $^{13}$C NMR Spectrum of **20** (101 MHz, $CDCl_3$)

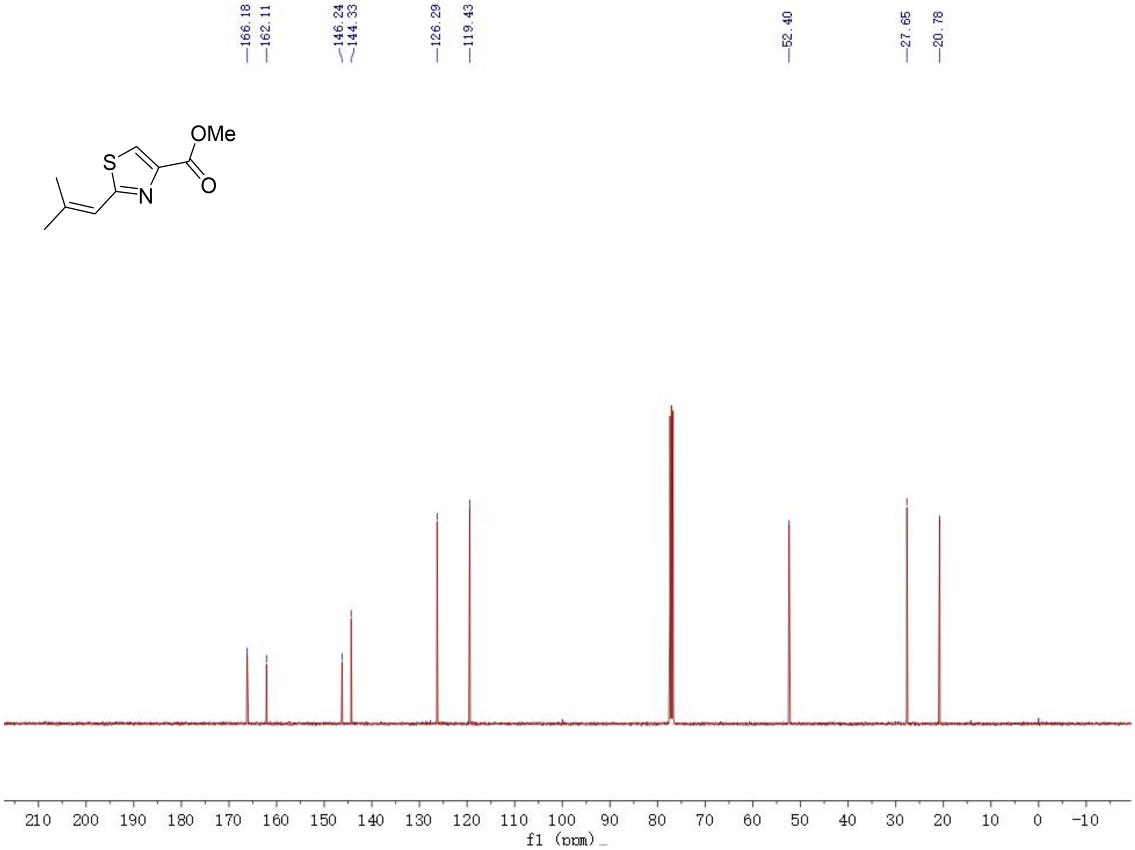

**Figures S9** $^{1}$H NMR Spectrum of **12** (400 MHz, $CDCl_3$)

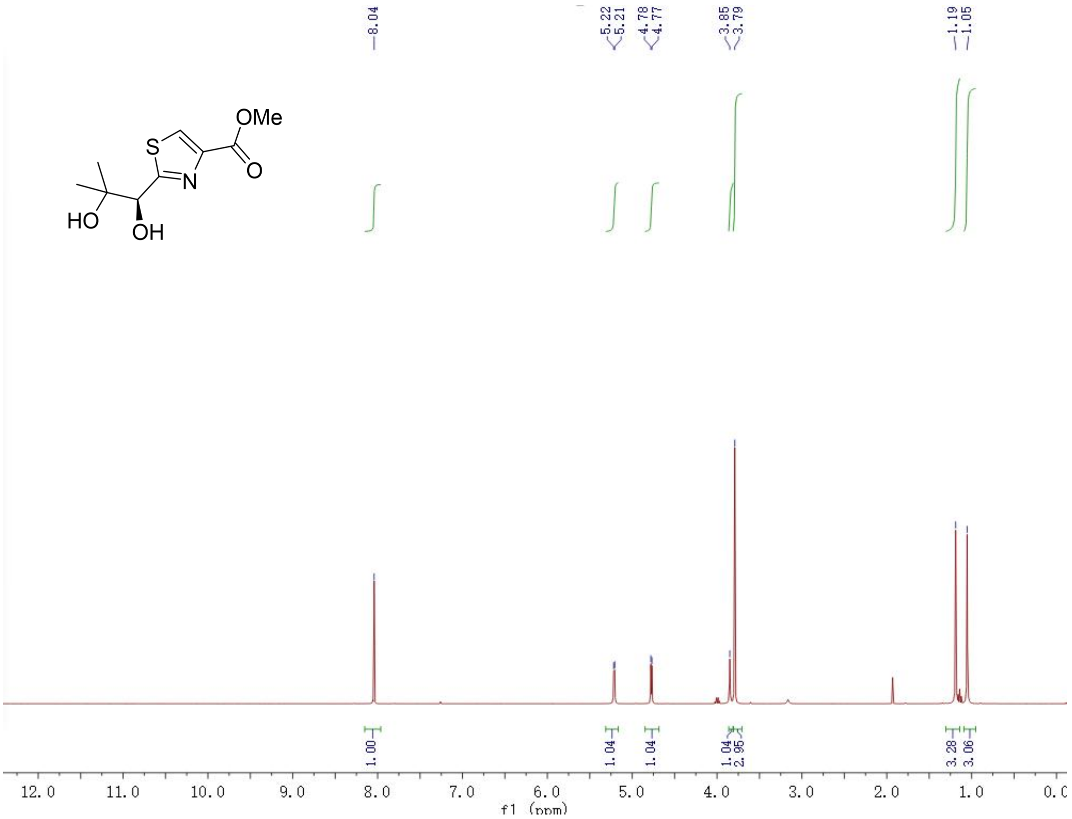


**Figures S10** $^{13}$C NMR Spectrum of **12** (101 MHz, $CDCl_3$)

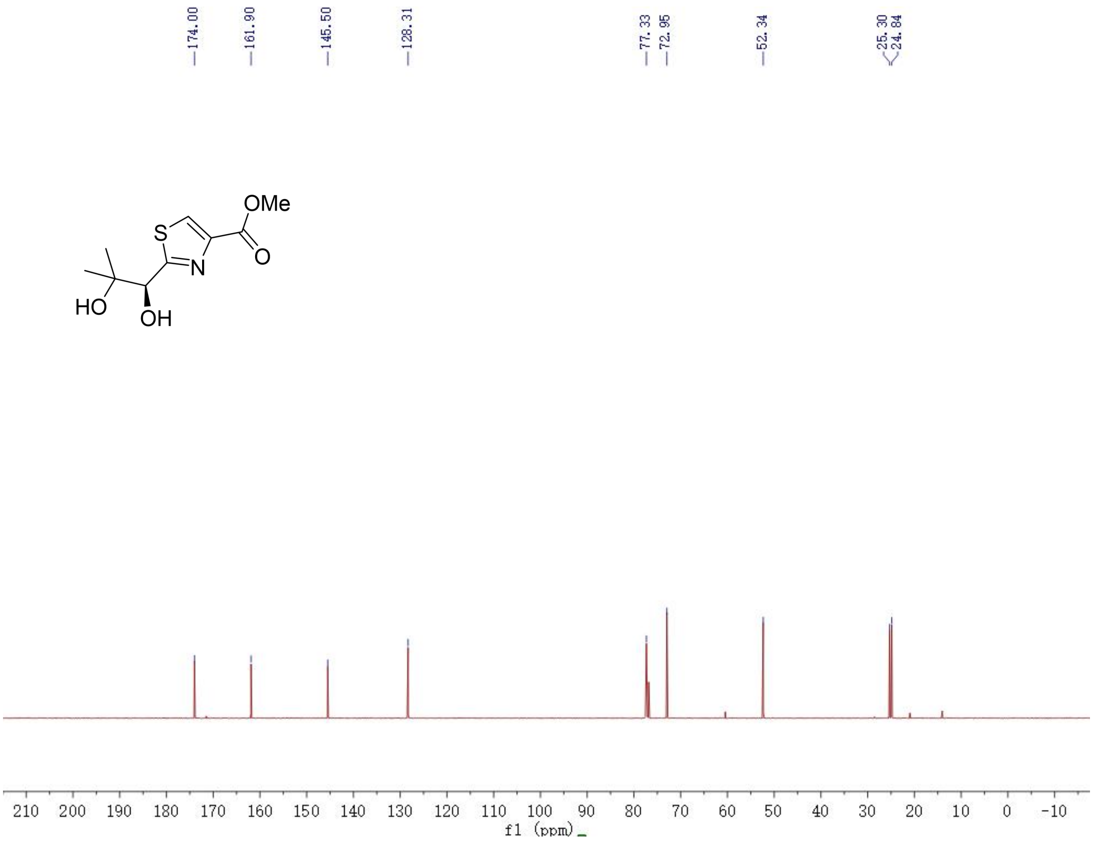

**Figures S11** $^{1}$H NMR Spectrum of **23** (400 MHz, $CDCl_3$)

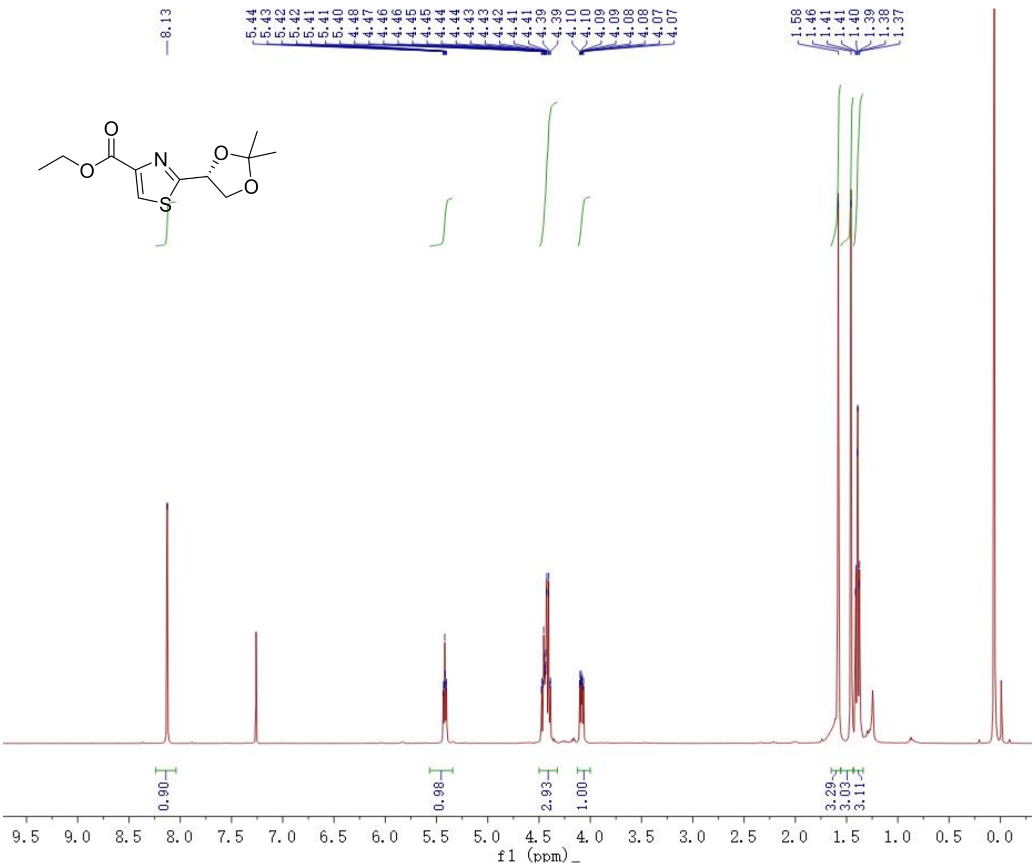


**Figures S12** $^{13}$C NMR Spectrum of **23** (101 MHz, $CDCl_3$)

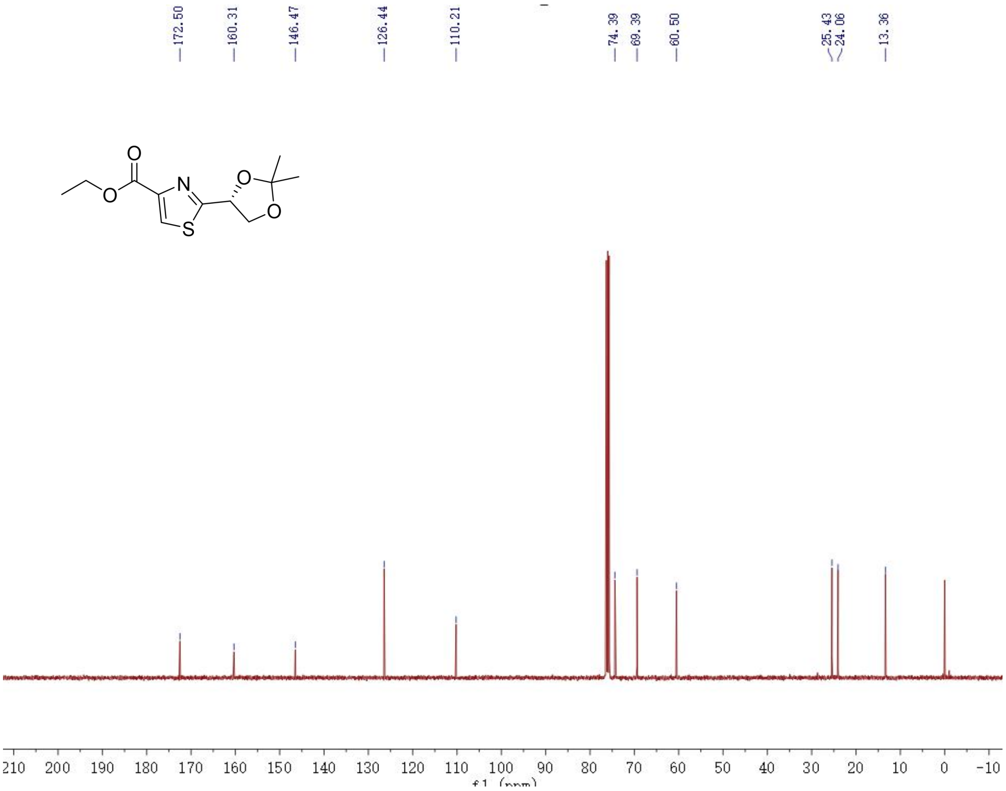

**Figures S13** $^{1}$H NMR Spectrum of **13** (400 MHz, $CDCl_3$)

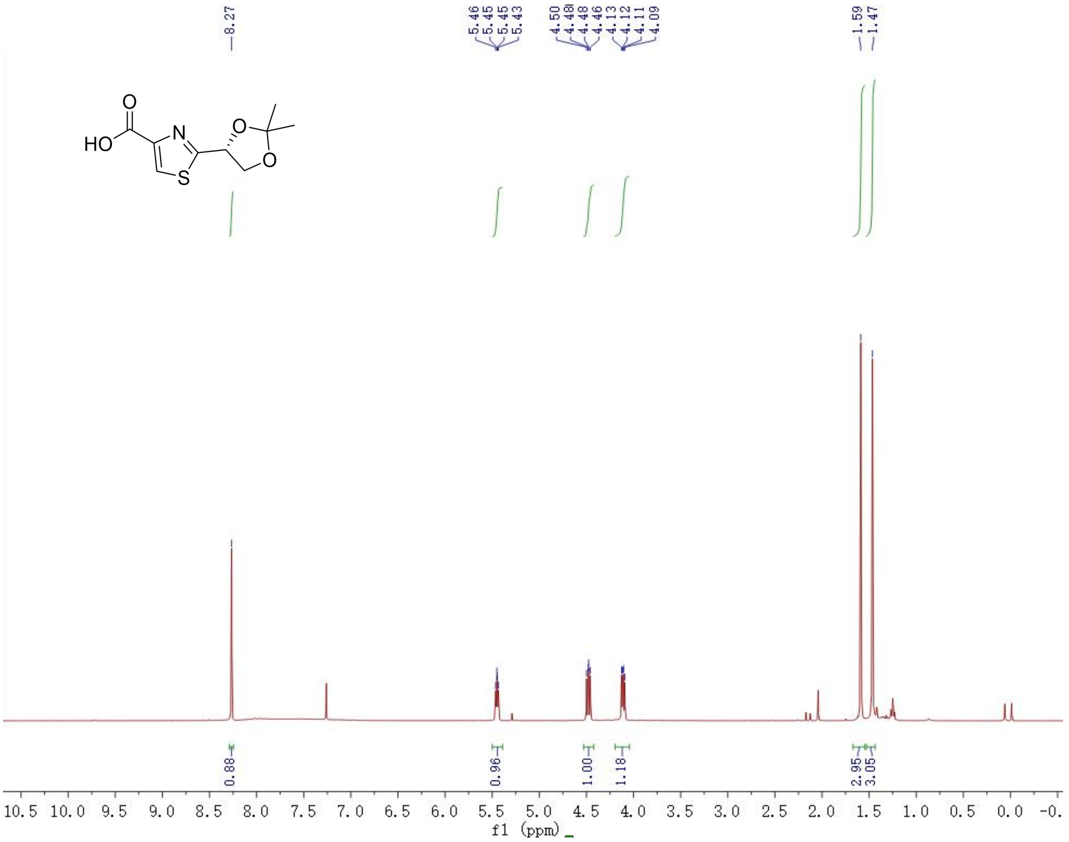


**Figures S14** $^{13}$C NMR Spectrum of **13** (101 MHz, $CDCl_3$)

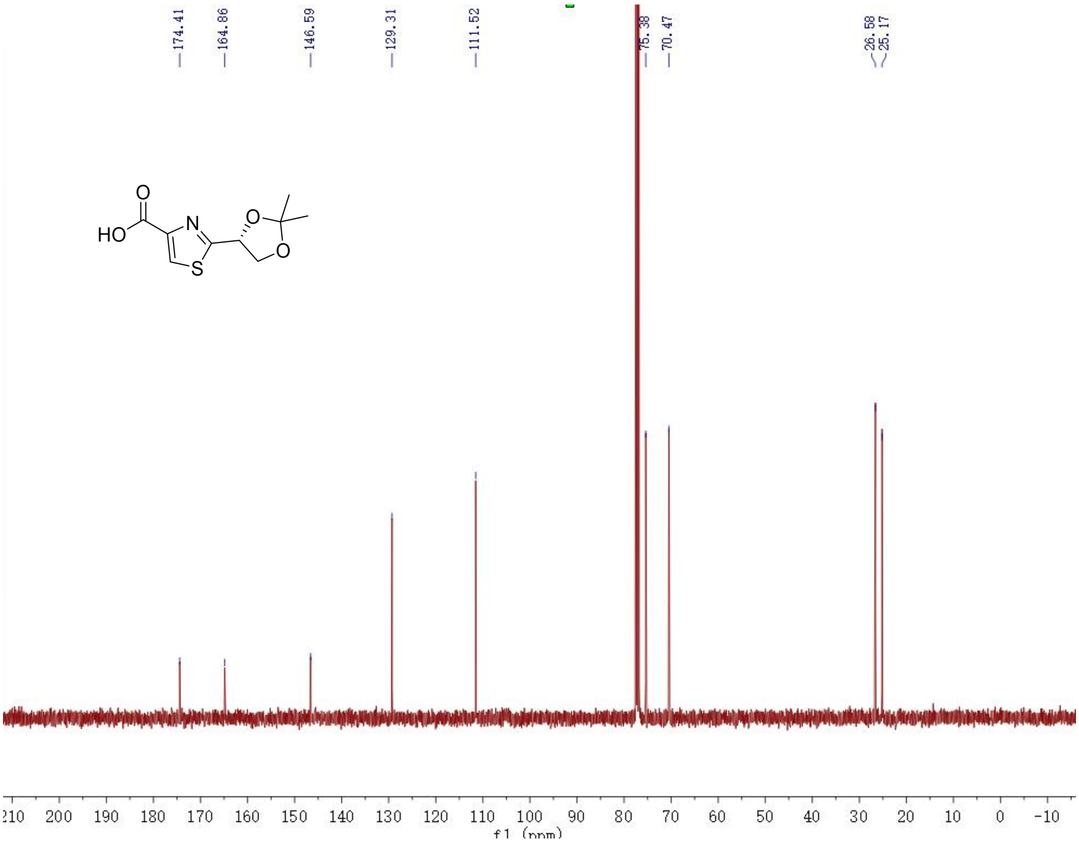

**Figures S15** $^{1}$H NMR Spectrum of **29** (400 MHz, $CDCl_3$)

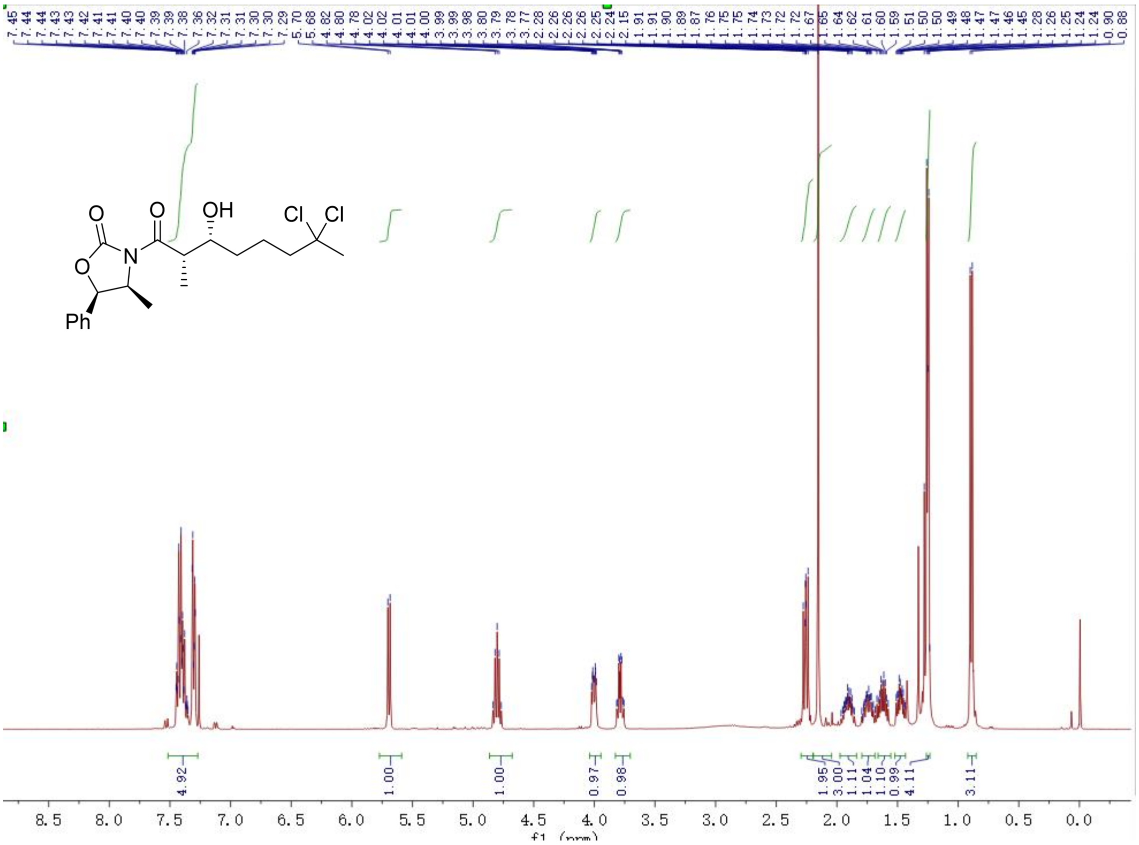


**Figures S16** $^{13}$C NMR Spectrum of **29** (101 MHz, $CDCl_3$)

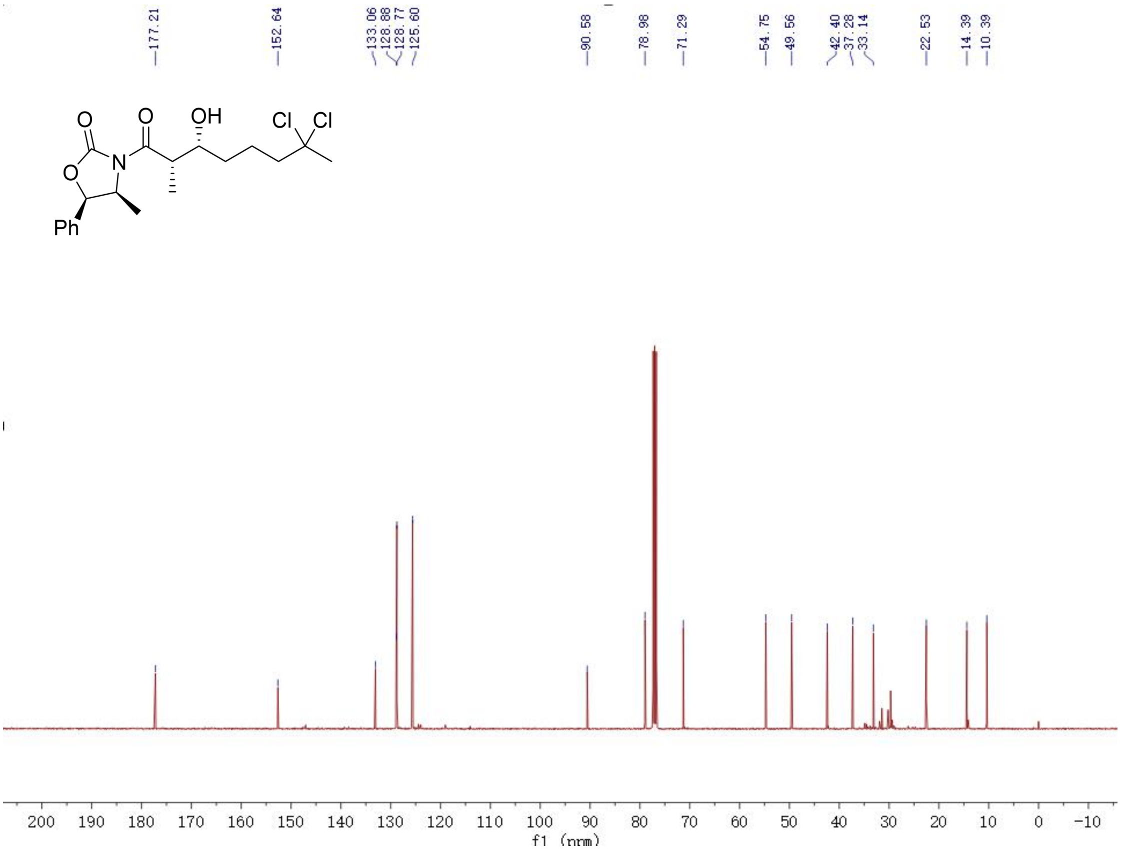

**Figures S17** $^{1}$H NMR Spectrum of **14** (400 MHz, $CDCl_3$)

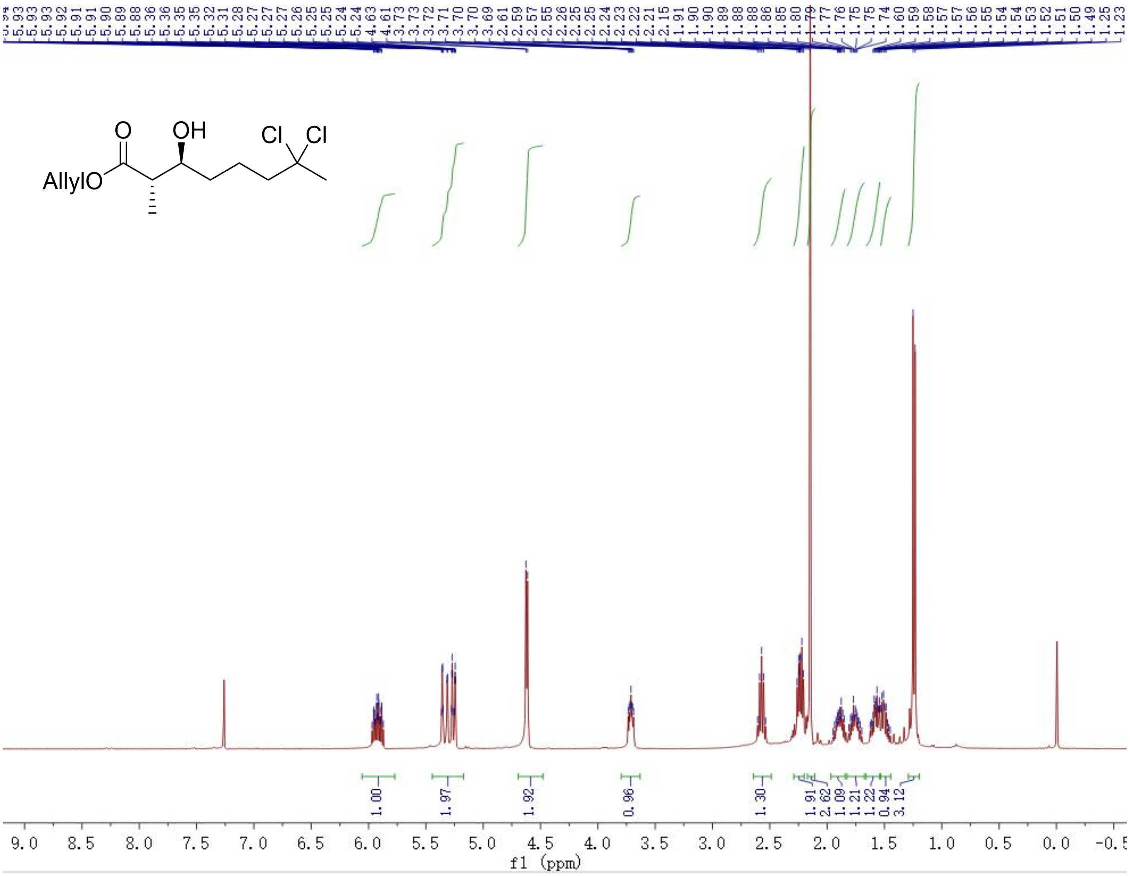

**Figures S18** $^{13}$C NMR Spectrum of **14** (101 MHz, $CDCl_3$)

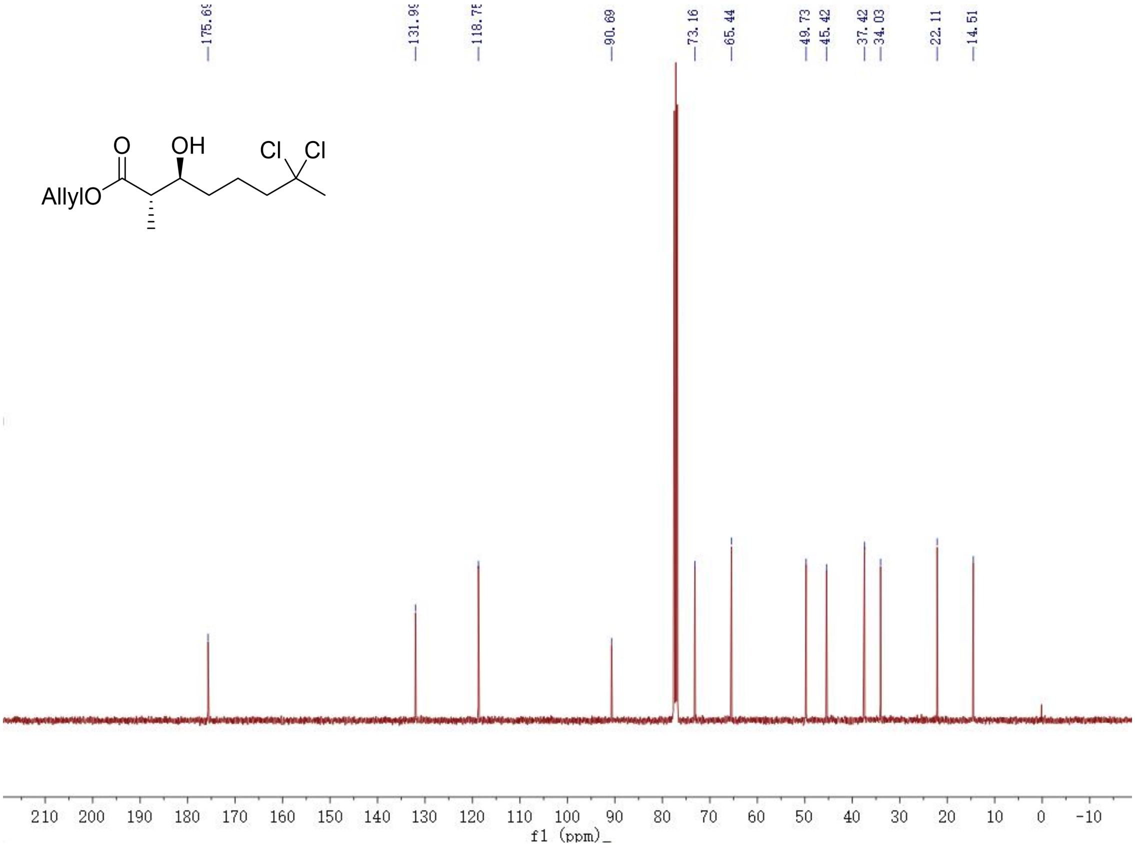

**Figures S19** $^{1}$H NMR Spectrum of **33** (400 MHz, $CDCl_3$)

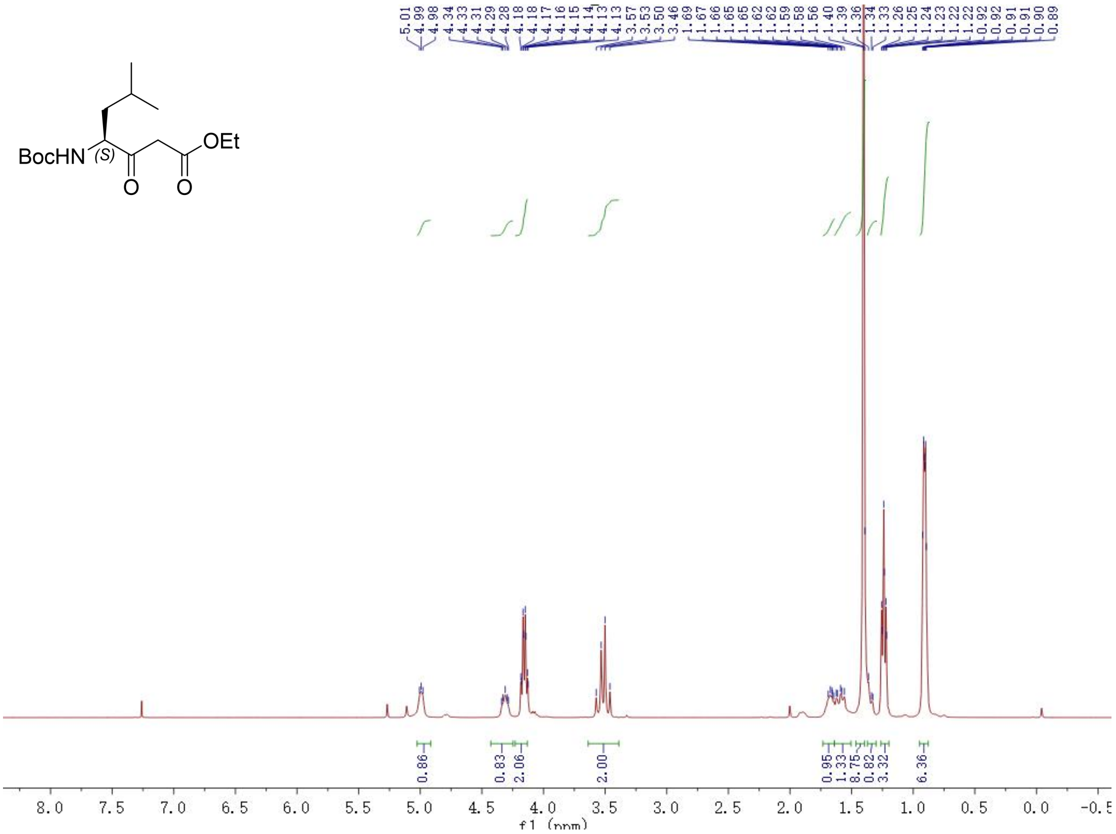


**Figures S20** $^{13}$C NMR Spectrum of **33** (101 MHz, $CDCl_3$)

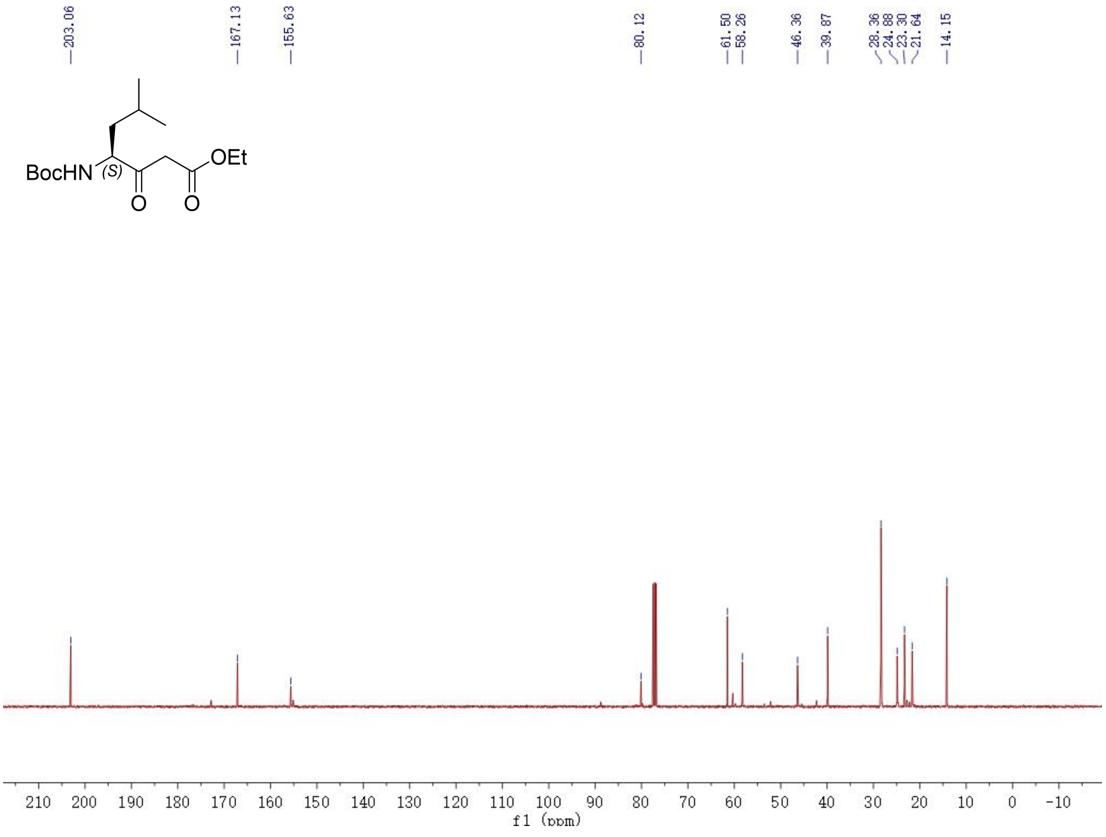

**Figures S21** $^{1}$H NMR Spectrum of **11** (400 MHz, CDCl$_3$)

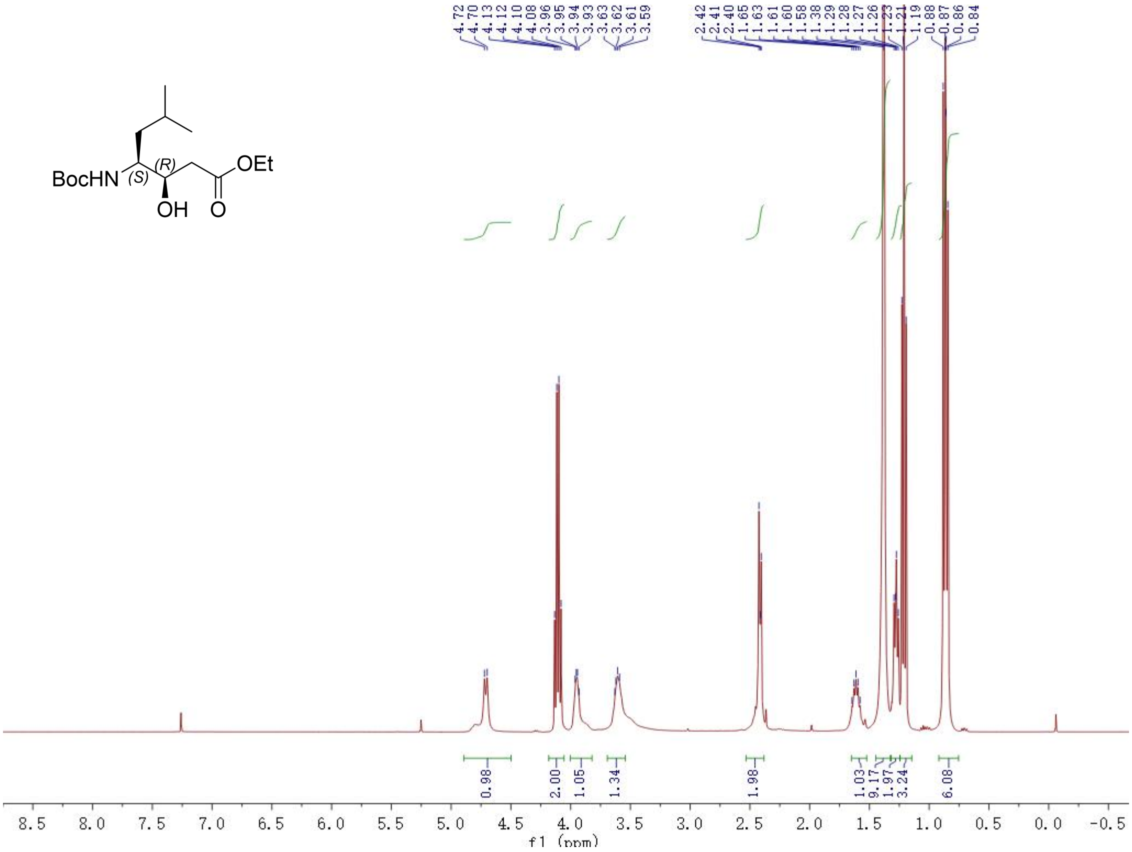


**Figures S22** $^{13}$C NMR Spectrum of **11** (101 MHz, CDCl$_3$)

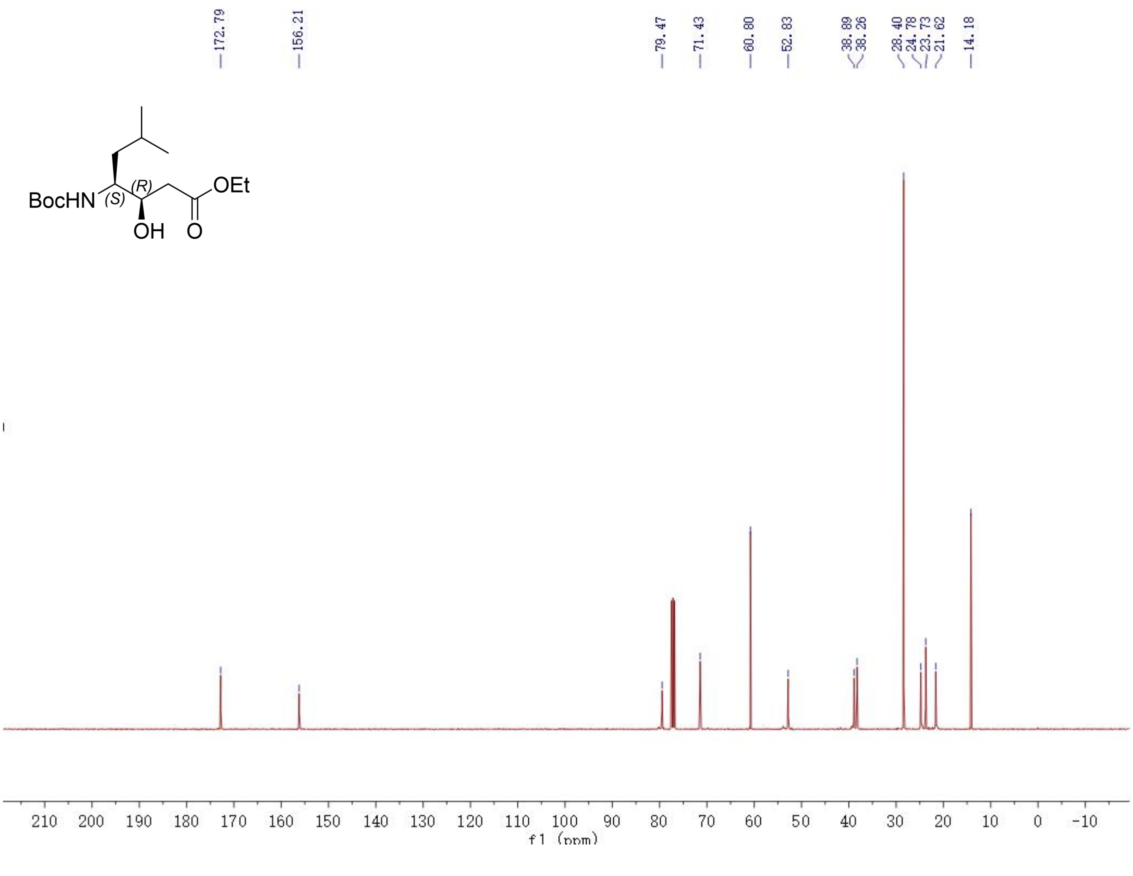

**Figures S23** $^{1}$H NMR Spectrum of **34** (400 MHz, $CDCl_3$)

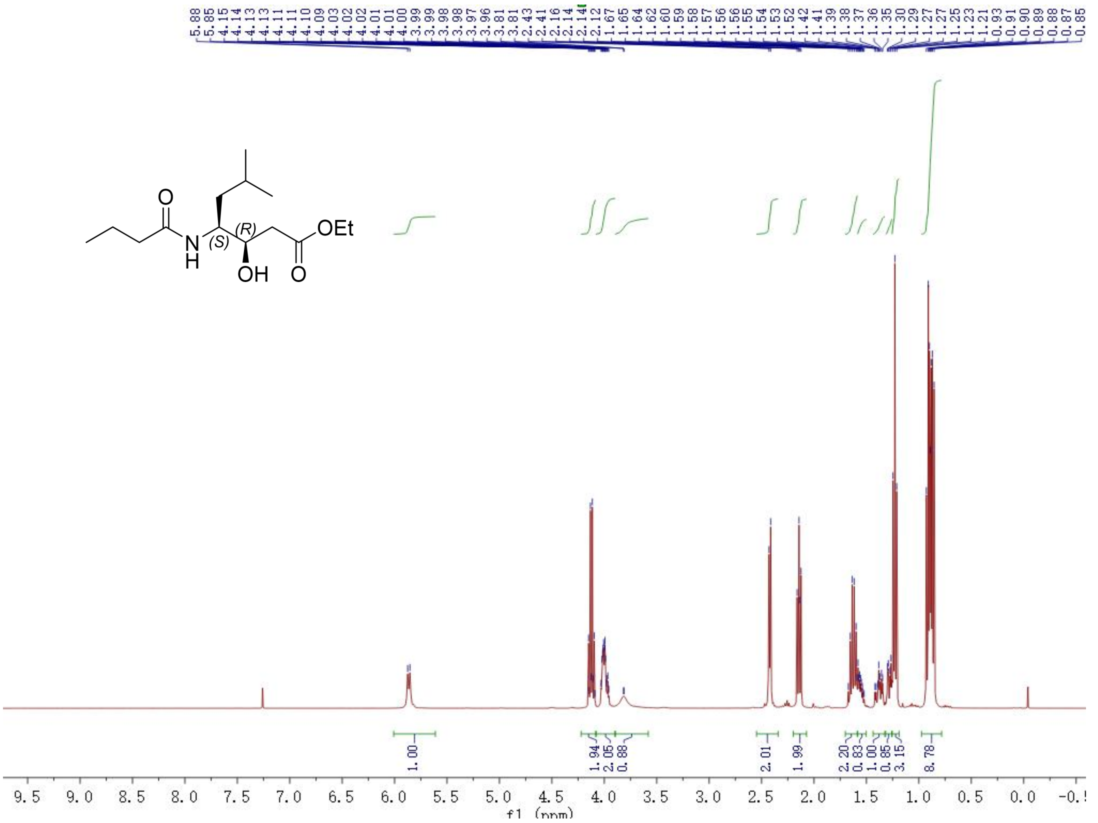

**Figures S24** $^{13}$C NMR Spectrum of **34** (101 MHz, $CDCl_3$)

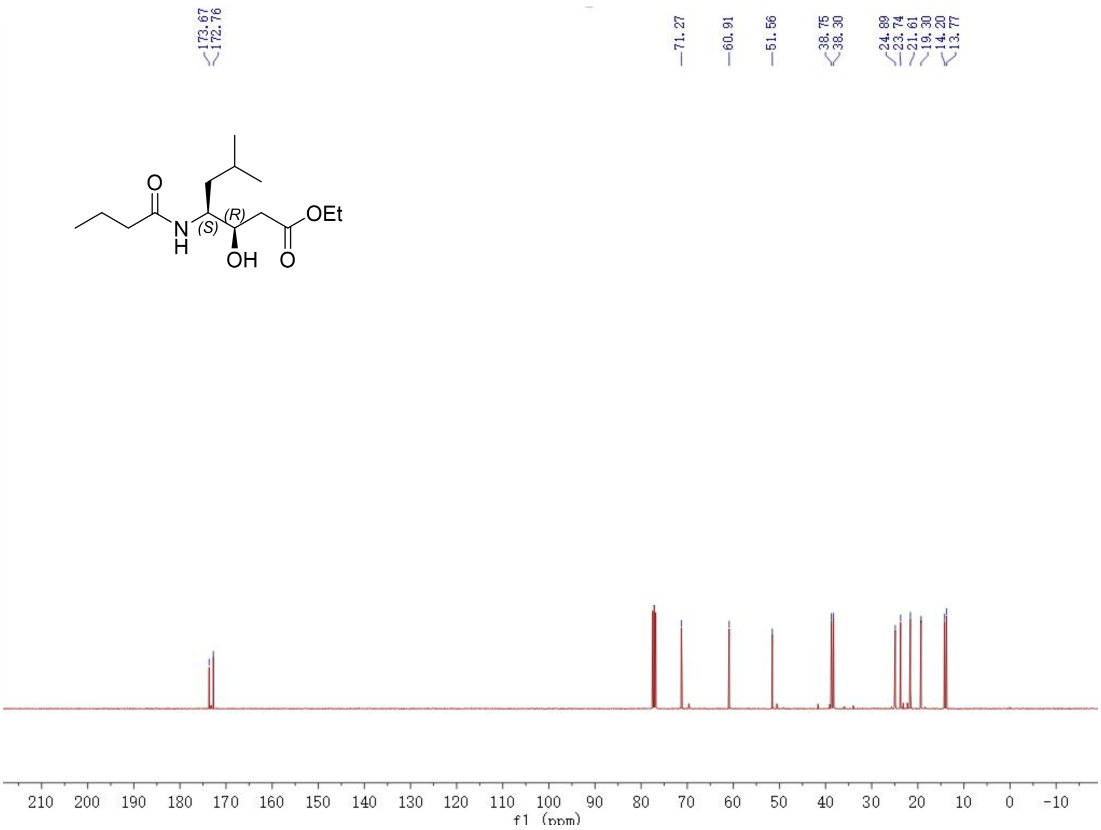

**Figures S25** $^{1}H$ NMR Spectrum of **10** (400 MHz, $CDCl_3$)

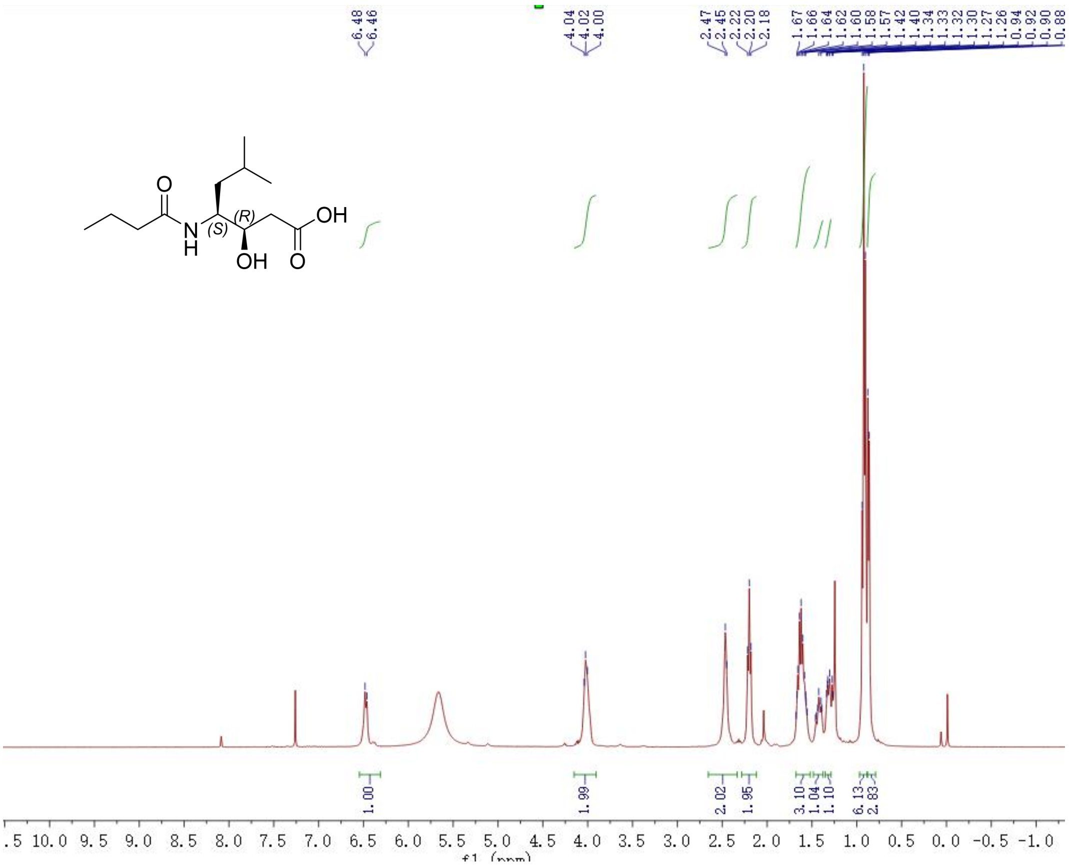

**Figures S26** $^{13}C$ NMR Spectrum of **10** (101 MHz, $CDCl_3$)

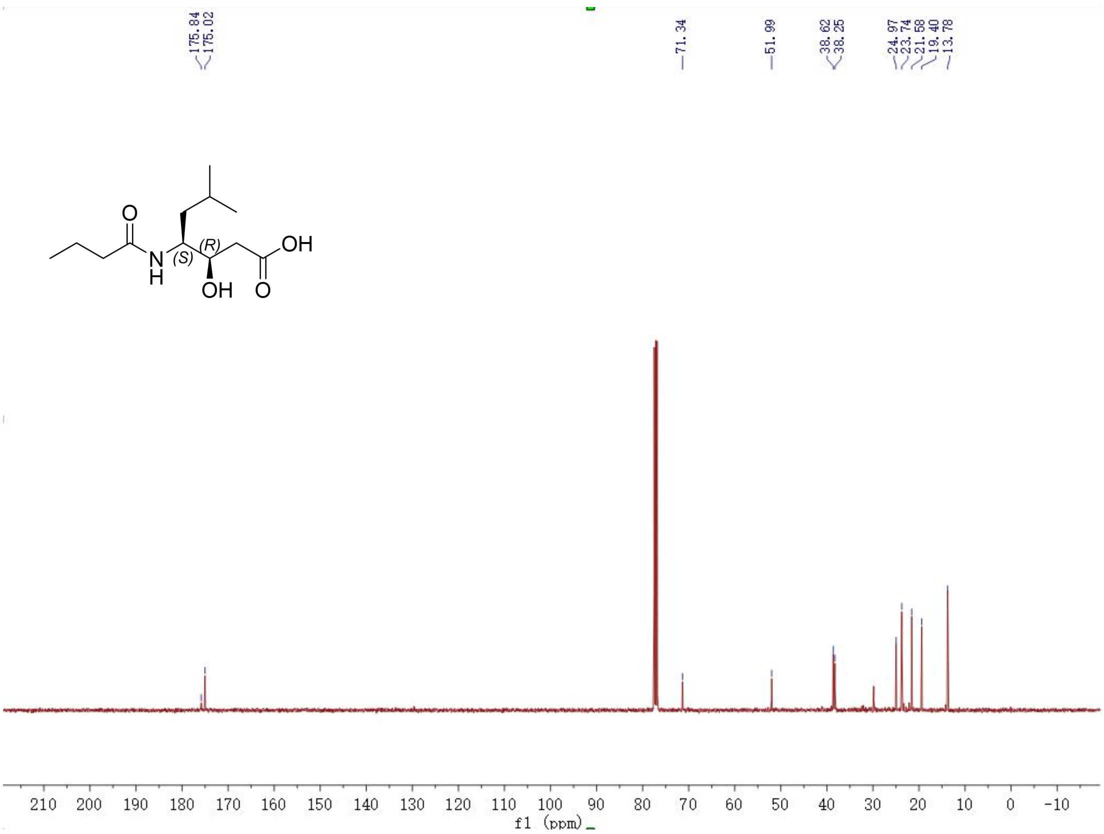

**Figures S27** $^{1}H$ NMR Spectrum of **35** (400 MHz, $CDCl_3$)

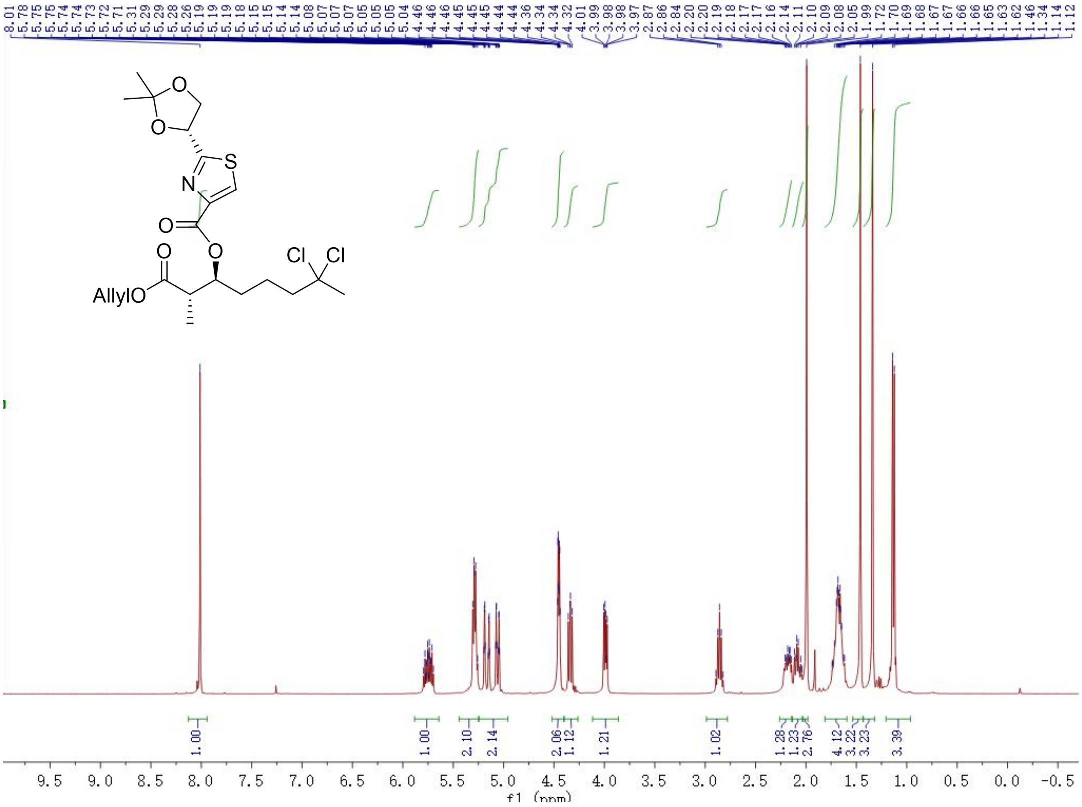

**Figures S28** $^{13}C$ NMR Spectrum of **35** (101 MHz, $CDCl_3$)

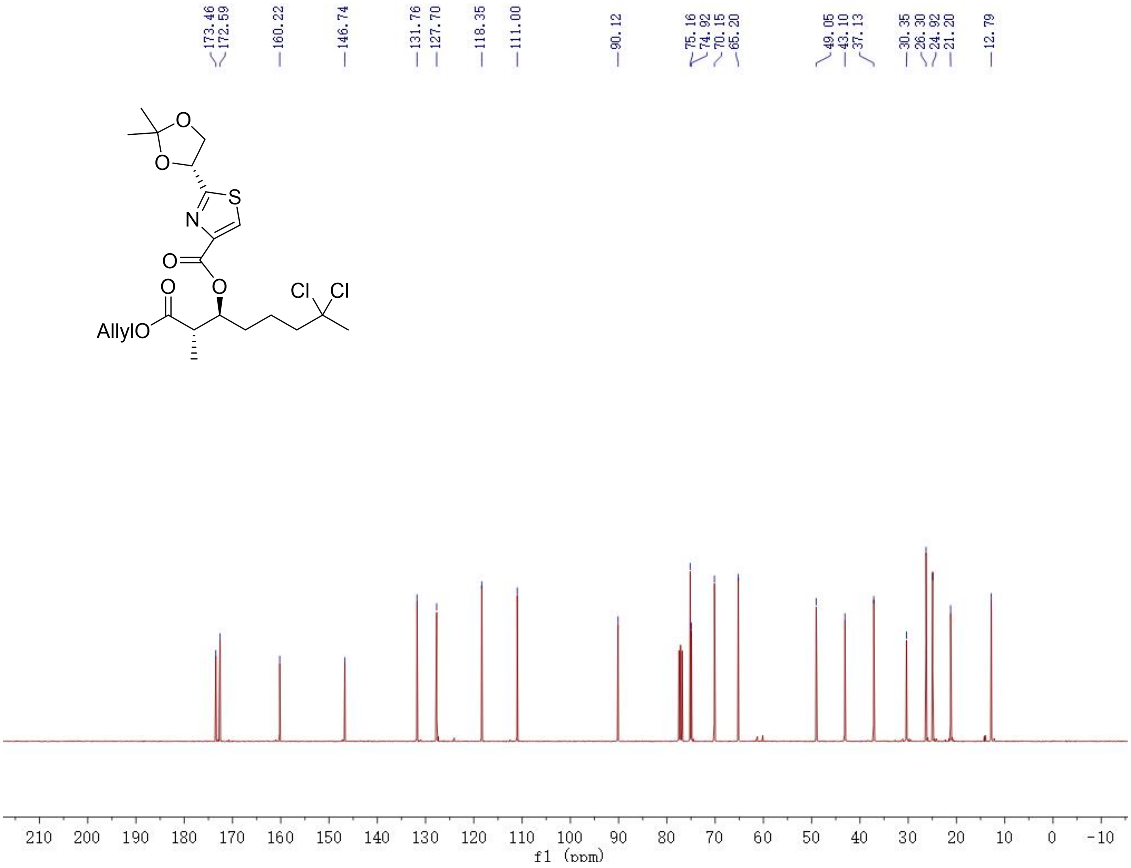

**Figures S29** $^{1}$H NMR Spectrum of **36** (400 MHz, $CDCl_3$)

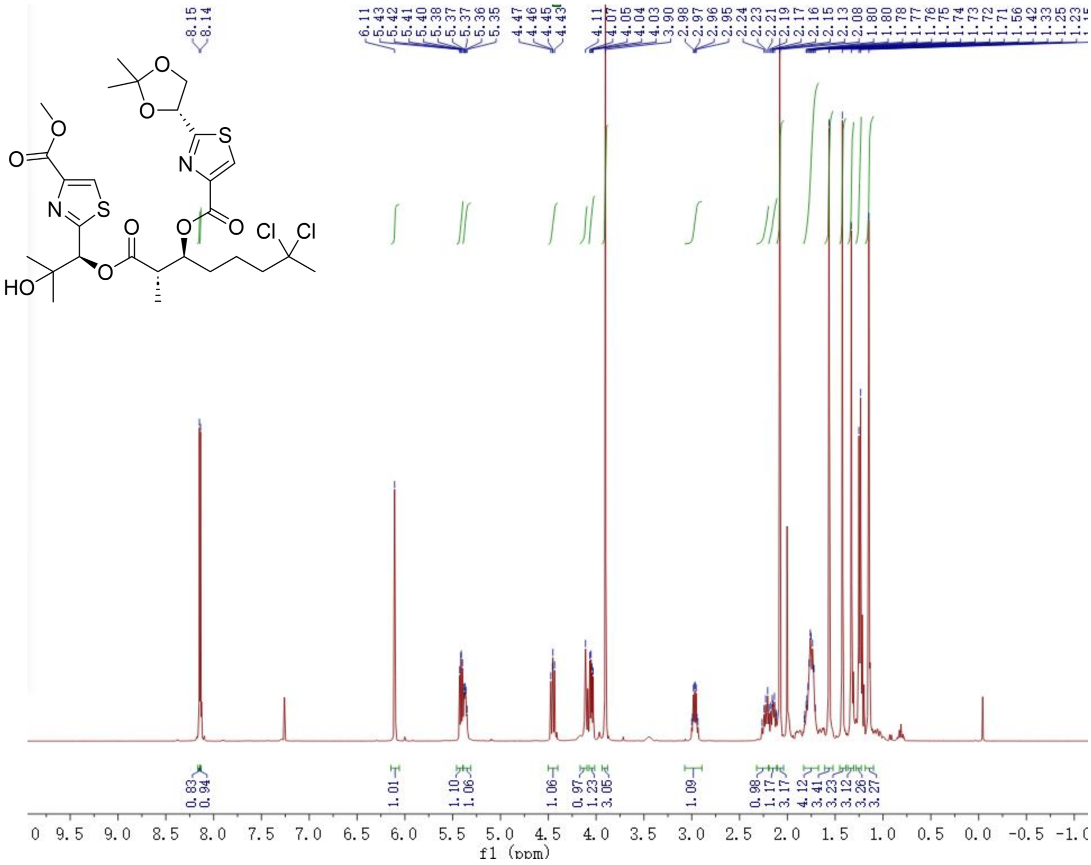


**Figures S30** $^{13}$C NMR Spectrum of **36** (101 MHz, $CDCl_3$)

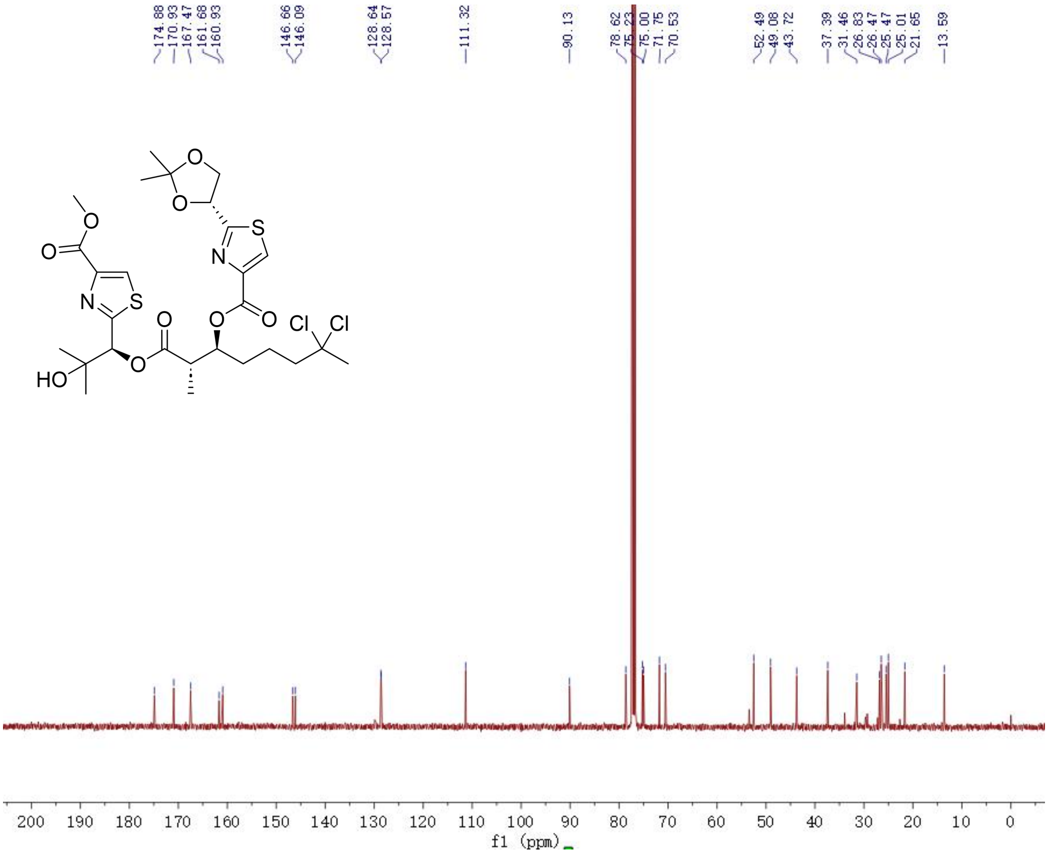

**Figures S31** $^1$H NMR Spectrum of lyngbyabellin O (**3**) (400 MHz, $CDCl_3$)

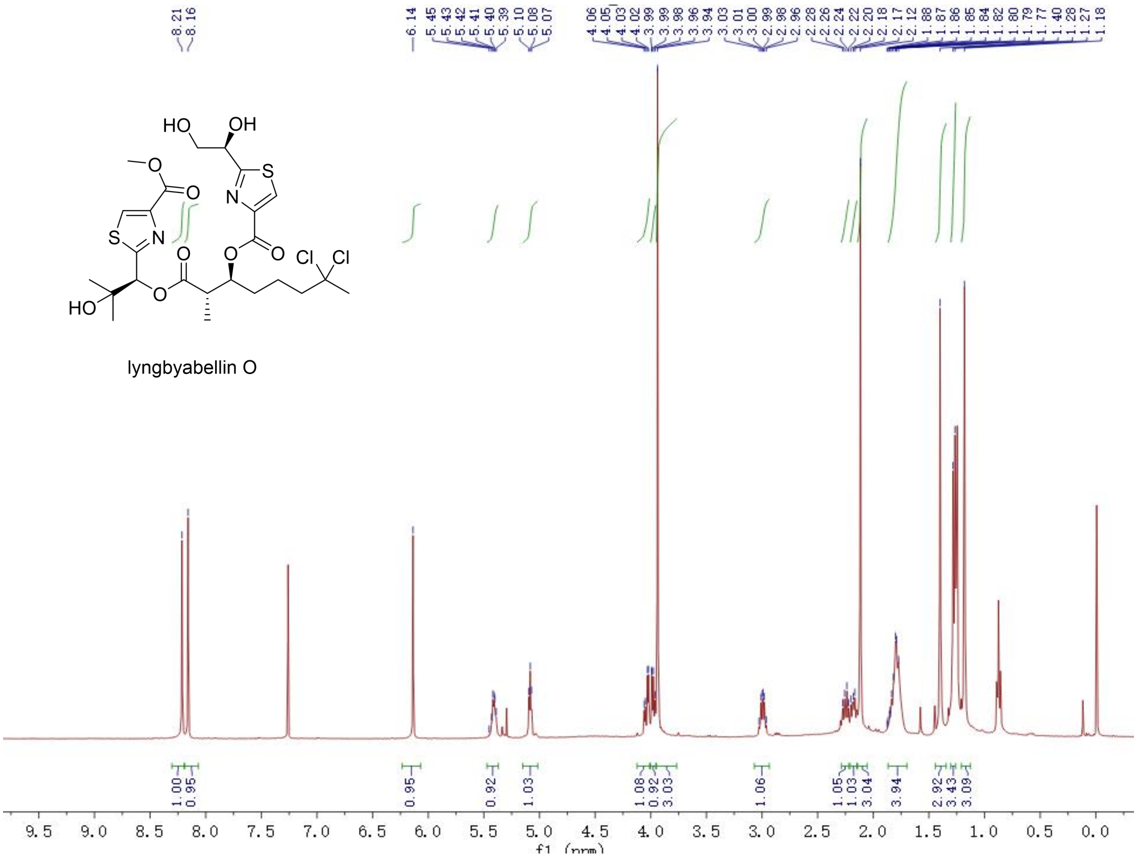


**Figures S32** $^{13}$C NMR Spectrum of lyngbyabellin O (**3**) (101 MHz, $CDCl_3$)

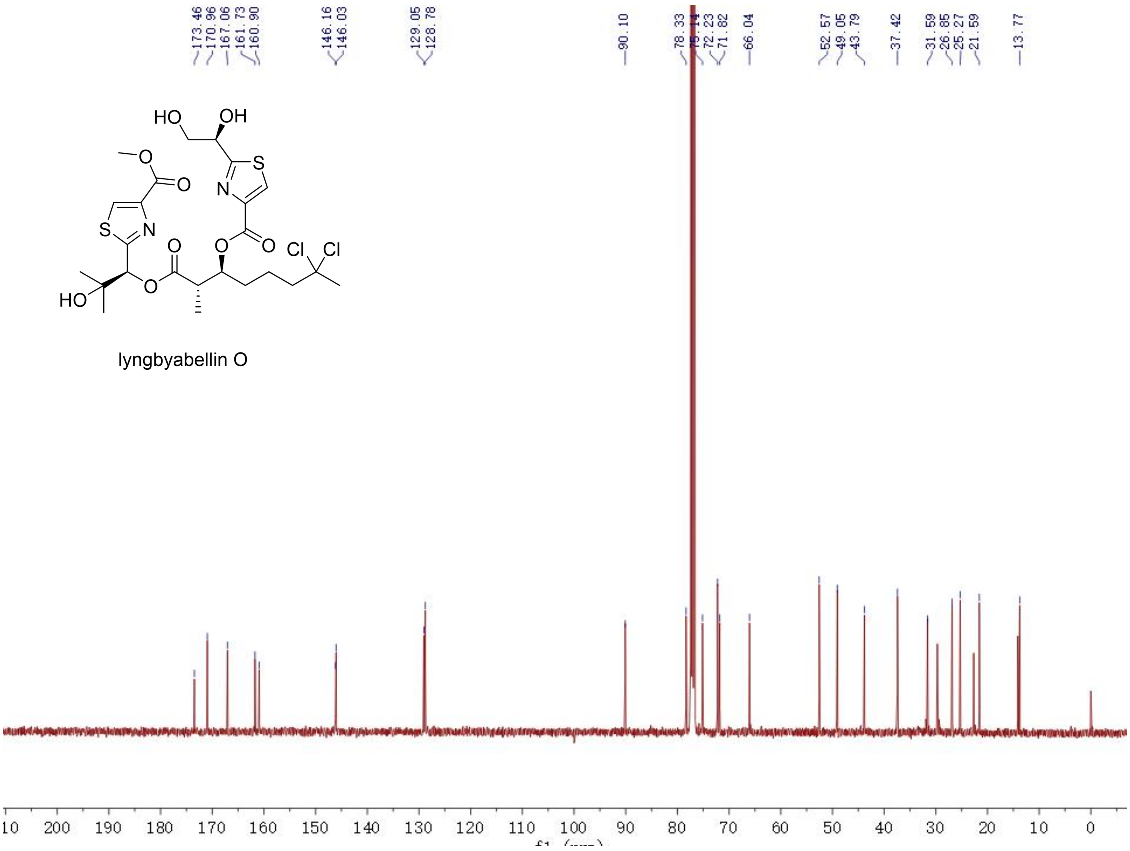

**Figures S33** $^{1}$H NMR Spectrum of lyngbyabellin P (**4**) (400 MHz, $CDCl_3$)

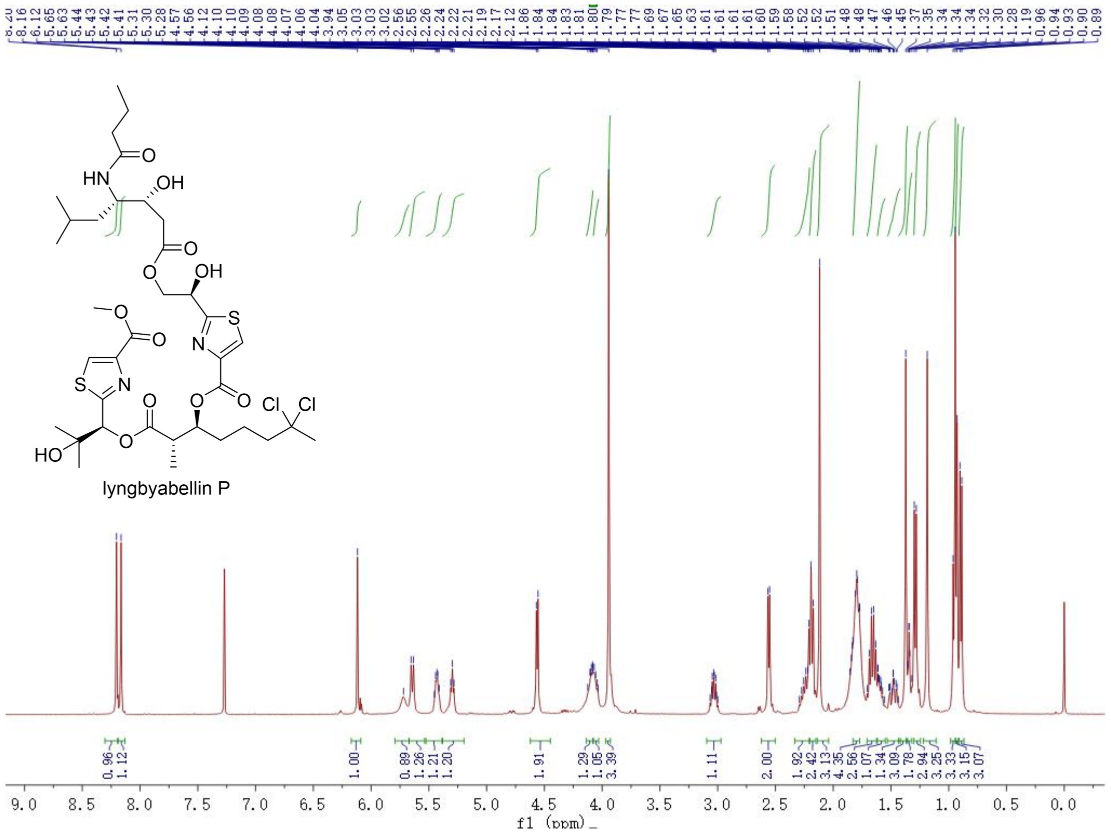


**Figures S34** $^{13}$C NMR Spectrum of lyngbyabellin P (**4**) (101 MHz, $CDCl_3$)

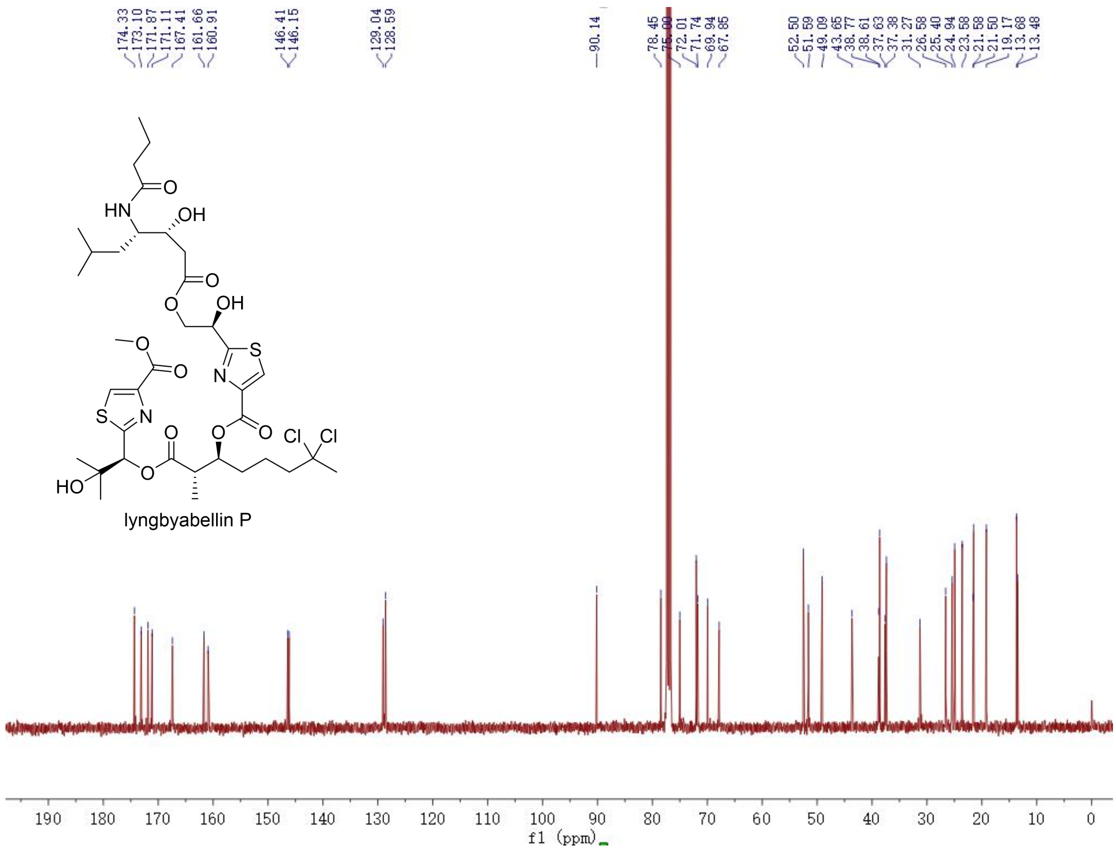

4. 2D NMR correlations for the structures of synthetic lyngbyabellin O and lyngbyabellin P

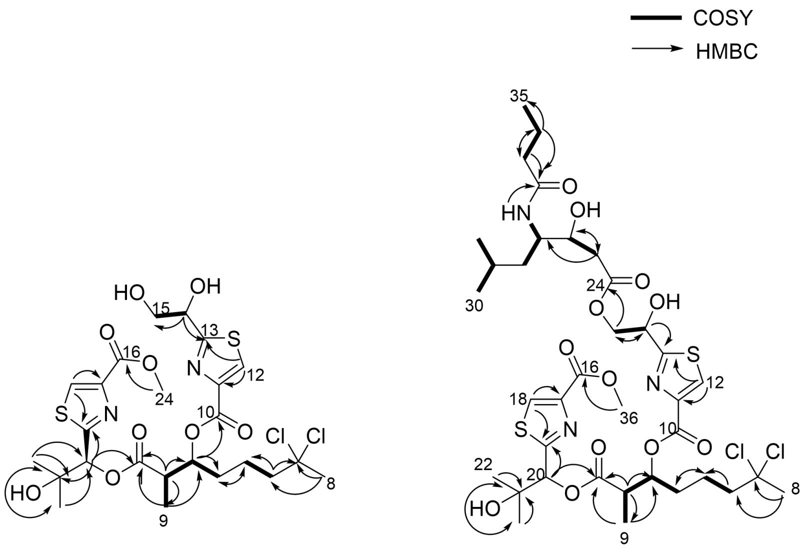


lyngbyabellin O (3) lyngbyabellin P (4)

**Figures S35** COSY spectrum of lyngbyabellin O (**3**) (400 MHz, CDCl3)

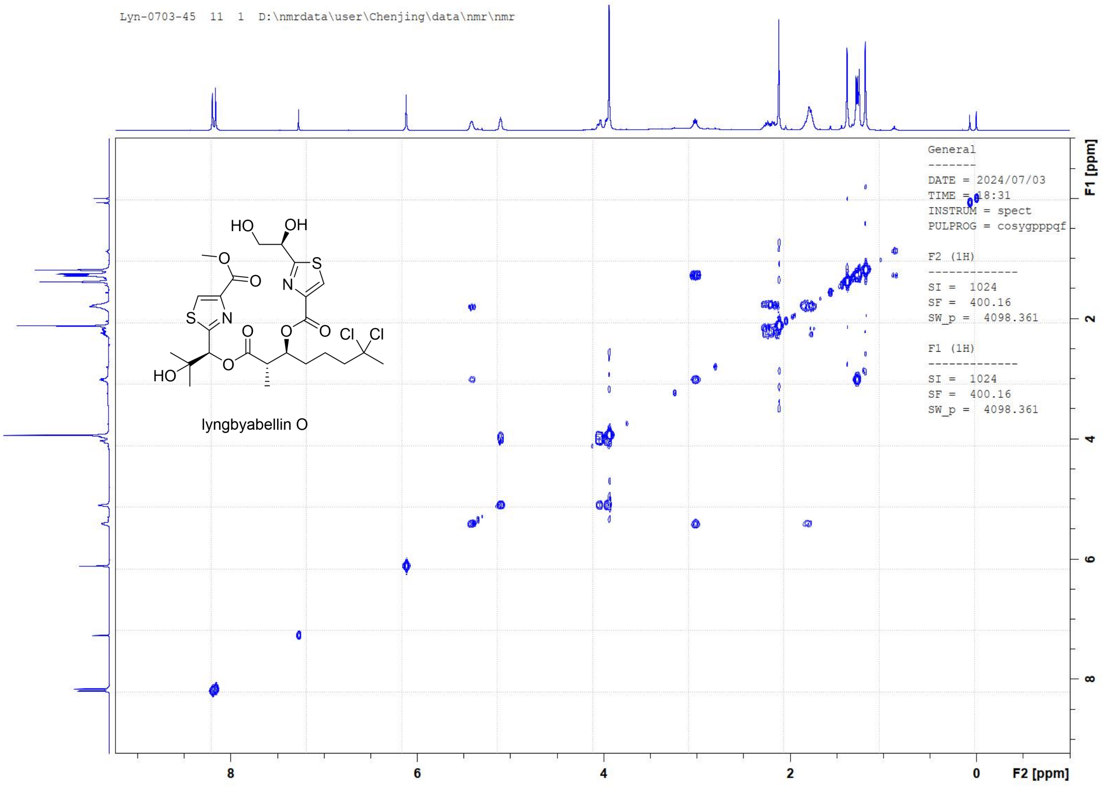

**Figures S36** HSQC spectrum of lyngbyabellin O (**3**) (400 MHz, $CDCl_3$)

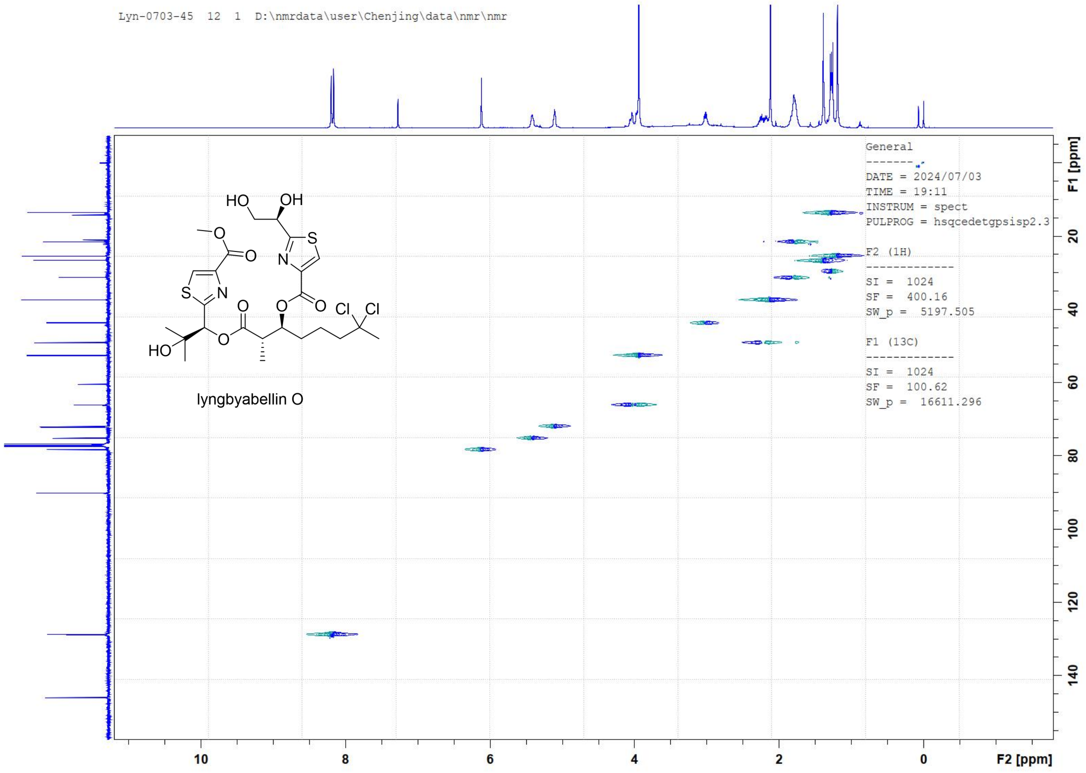


**Figures S37** HMBC spectrum of lyngbyabellin O (**3**) (400 MHz, $CDCl_3$)

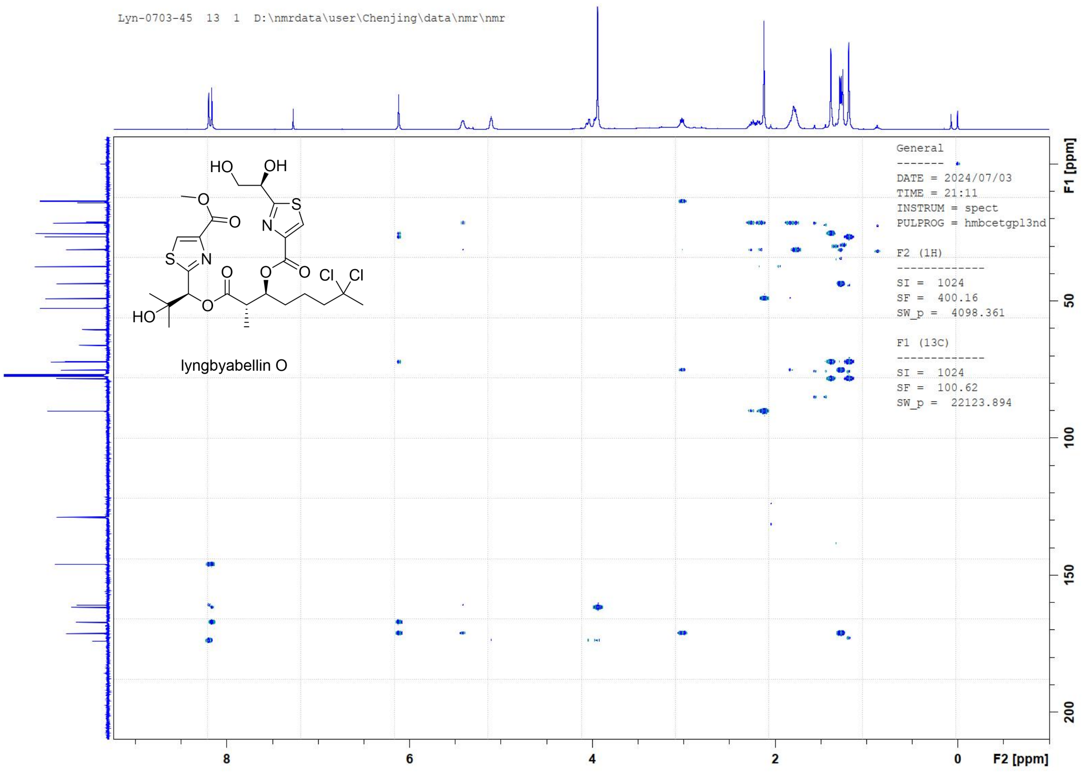

**Figures S38** COSY spectrum of lyngbyabellin P (**4**) (400 MHz, $CDCl_3$)

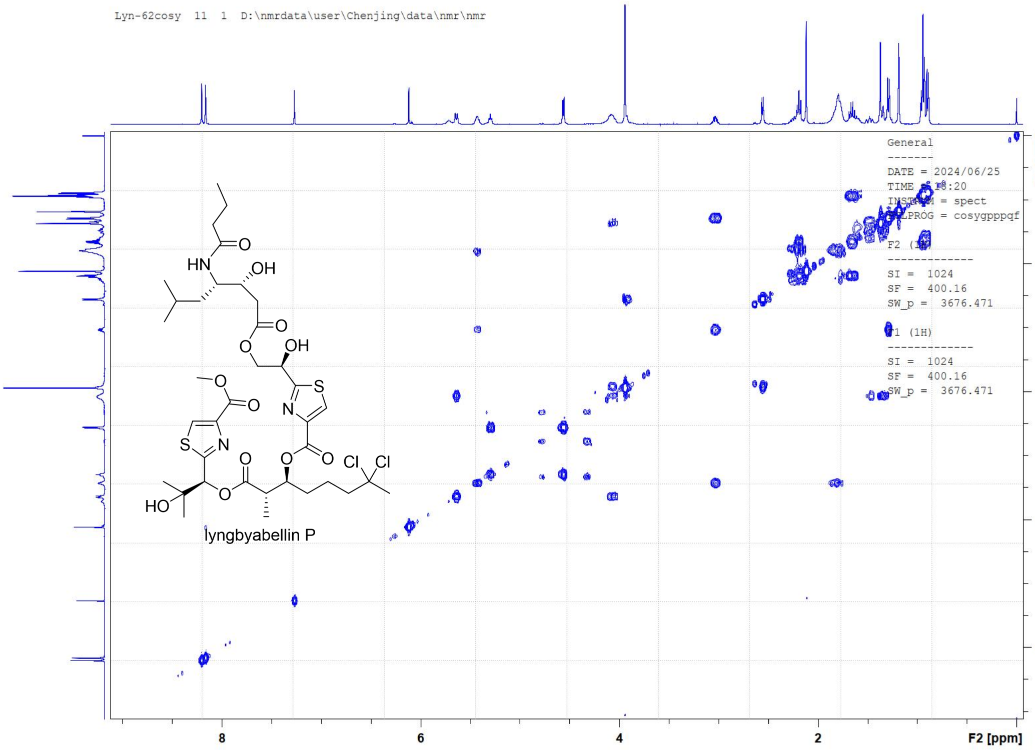


**Figures S39** HSQC spectrum of lyngbyabellin P (**4**) (400 MHz, $CDCl_3$)

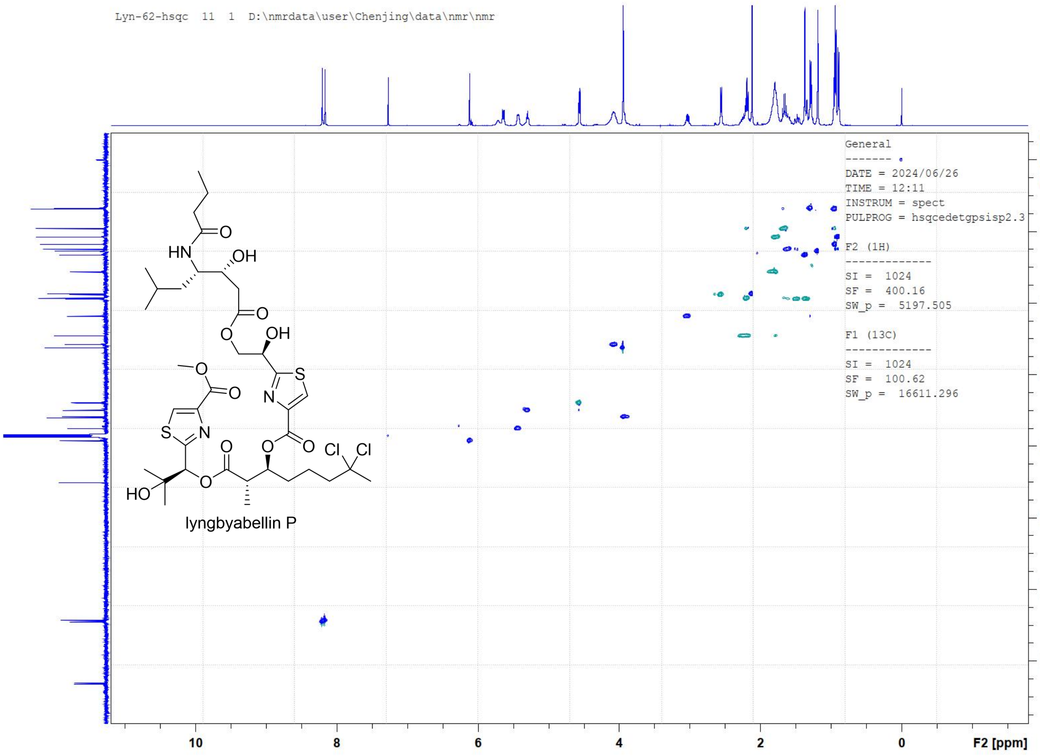

**Figures S40** HMBC spectrum of lyngbyabellin P (**4**) (400 MHz, $CDCl_3$)

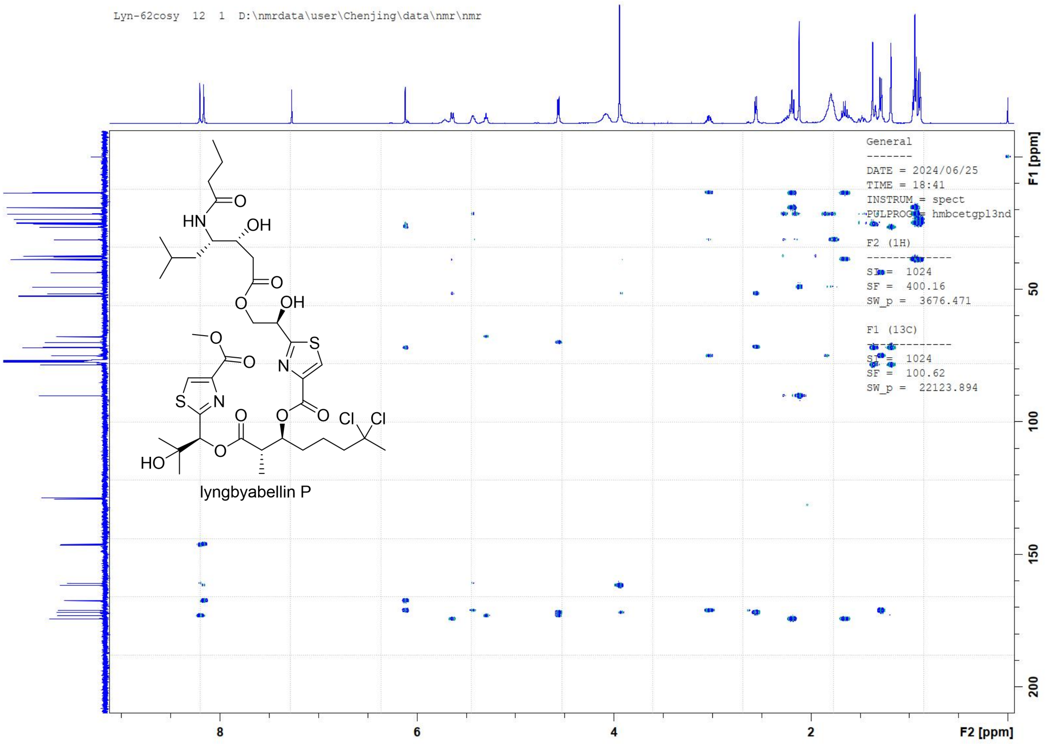

5. Comparative $^{13}C$ NMR spectra of natural and synthetic lyngbyabellin O and lyngbyabellin P

**Figures S41** Comparative $^{13}C$ NMR spectra of natural and synthetic lyngbyabellin O (natural in top, synthetic in bottom)

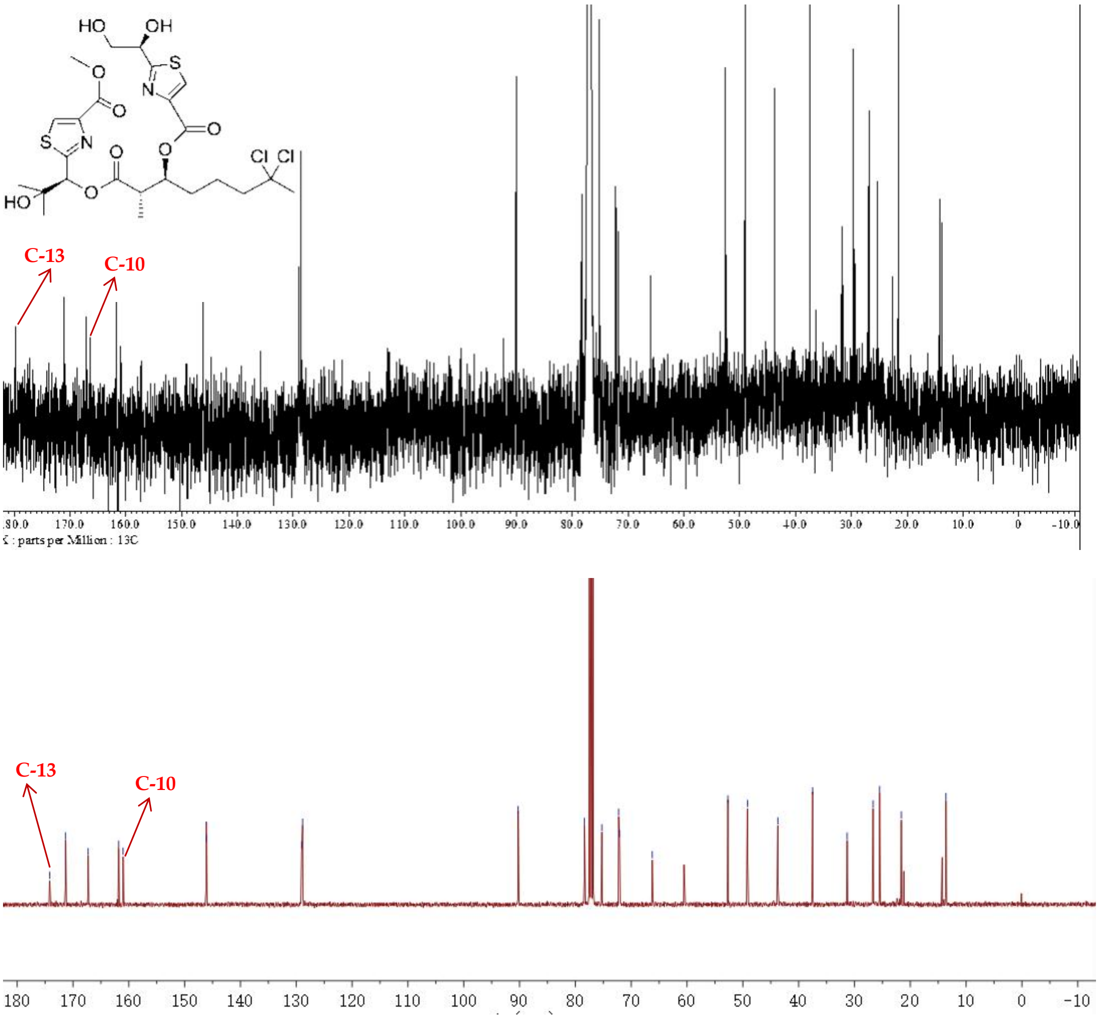

**Figures S42** Comparative $^{13}$C NMR spectra of natural and synthetic lyngbyabellin P (natural in top, synthetic in bottom)

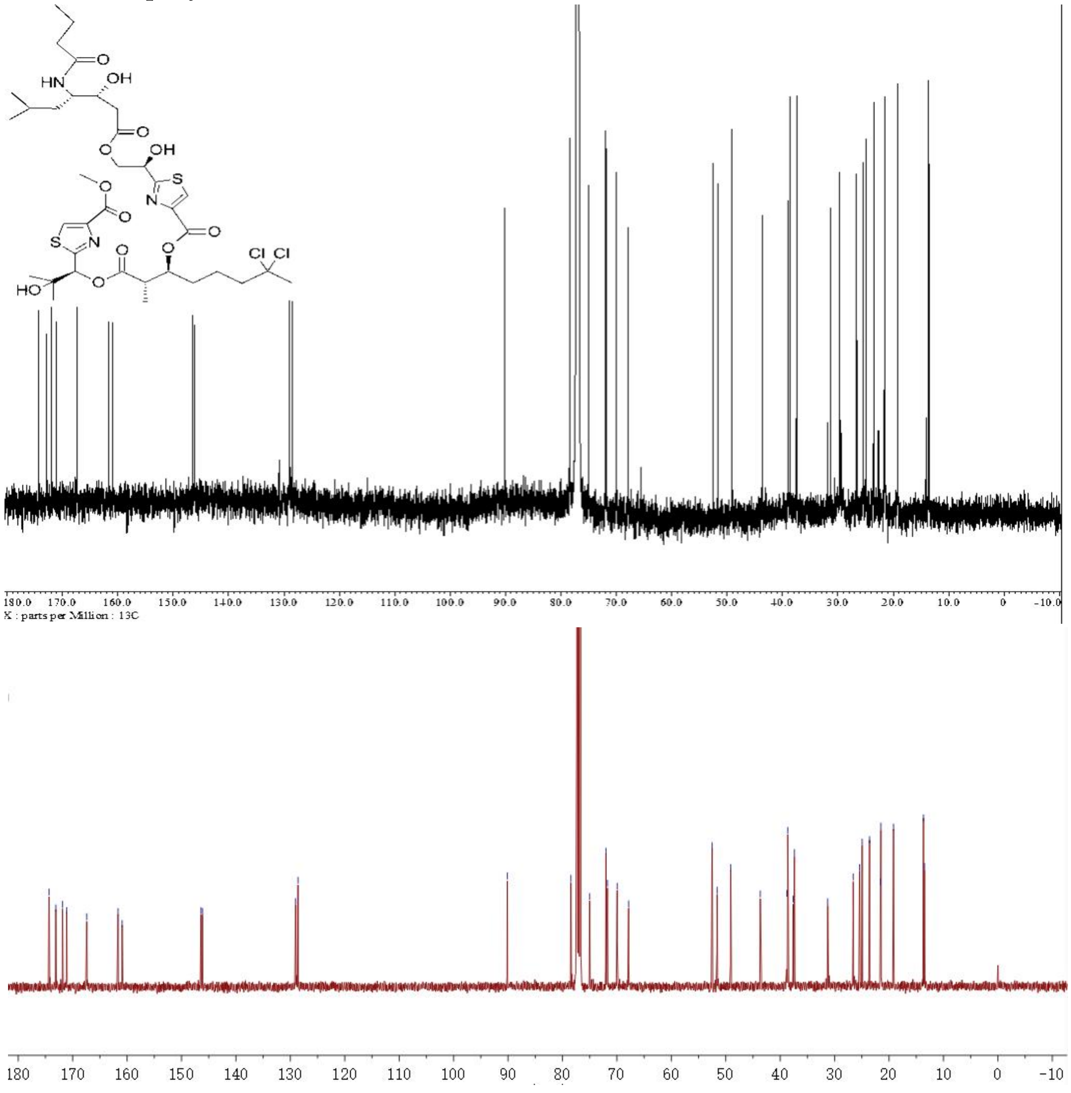

Supplement: Supplementary file 1 [file marinedrugs-23-00340-s001.zip › marinedrugs-3821346-supplementary.pdf]
